# A mannose-sensing AraC-type transcriptional activator regulates cell–cell aggregation of *Vibrio cholerae*

**DOI:** 10.1038/s41522-022-00331-x

**Published:** 2022-08-20

**Authors:** Hye-Young Lee, Chang-Kyu Yoon, Yong-Joon Cho, Jin-Woo Lee, Kyung-Ah Lee, Won-Jae Lee, Yeong-Jae Seok

**Affiliations:** 1 Department of Biophysics and Chemical Biology, Seoul National University, Seoul, 08826 Republic of Korea; 2 School of Biological Sciences and Institute of Microbiology, Seoul National University, Seoul, 08826 Republic of Korea; 3 School of Biological Sciences, Seoul National University, Seoul, 08826 Republic of Korea

**Keywords:** Microbial genetics, Biofilms, Pathogens

## Abstract

In addition to catalyzing coupled transport and phosphorylation of carbohydrates, the phosphoenolpyruvate:carbohydrate phosphotransferase system (PTS) regulates various physiological processes in most bacteria. Therefore, the transcription of genes encoding the PTS is precisely regulated by transcriptional regulators depending on substrate availability. As the distribution of the mannose-specific PTS (PTS^Man^) is limited to animal-associated bacteria, it has been suggested to play an important role in host-bacteria interactions. In *Vibrio cholerae*, mannose is known to inhibit biofilm formation. During host infection, the transcription level of the *V. cholerae* gene encoding the putative PTS^Man^ (hereafter referred to as *manP*) significantly increases, and mutations in this gene increase host survival rate. Herein, we show that an AraC-type transcriptional regulator (hereafter referred to as ManR) acts as a transcriptional activator of the mannose operon and is responsible for *V. cholerae* growth and biofilm inhibition on a mannose or fructose-supplemented medium. ManR activates mannose operon transcription by facilitating RNA polymerase binding to the promoter in response to mannose 6-phosphate and, to a lesser extent, to fructose 1-phosphate. When *manP* or *manR* is impaired, the mannose-induced inhibition of biofilm formation was reversed and intestinal colonization was significantly reduced in a *Drosophila melanogaster* infection model. Our results show that ManR recognizes mannose and fructose in the environment and facilitates *V. cholerae* survival in the host.

## Introduction

*Vibrio cholerae* is a Gram-negative bacterium that belongs to the class Gammaproteobacteria. The natural habitat of *V. cholerae* is an aquatic environment such as an estuary or brackish water. In humans, *V. cholerae* infection of the intestines through water or food causes cholera, a fatal diarrheal disease^1,2^. Through various mechanisms to modify gene expression related to nutrient uptake, virulence, and biofilm formation, *V. cholerae* has evolved to effectively colonize disparate ecological niches across nutrient-rich human small intestines and aquatic environments^3^.

Most bacteria rely on the phosphoenolpyruvate (PEP):carbohydrate phosphotransferase system (PTS) to transport sugars from the environment into the cells by simultaneously phosphorylating them^4^. The PTS comprises two general components, enzyme I (EI) and histidine-containing phosphocarrier protein (HPr), which are commonly used to transport most PTS sugars, and various sugar-specific enzyme II (EII) complexes. Each EII complex usually comprises two cytoplasmic domains (EIIA and EIIB) and one transmembrane domain (EIIC). EI is autophosphorylated by PEP, and HPr mediates the phosphoryl transfer reaction from EI to EIIA. The phosphate group is then transferred to EIIB, where the PTS sugar is phosphorylated during transport into the cell via EIIC. In addition to its roles in carbohydrate transport and phosphorylation, the PTS acts as an efficient signaling system capable of regulating various cellular functions by sensing the availability of carbohydrates in the environment^4^. Therefore, genes encoding the PTS are also precisely regulated by the cooperative action of various mechanisms; this regulatory mechanism is important for bacterial survival^5^.

Mannose, a dominant monosaccharide in mucosal glycoproteins^6^, is an epimer of glucose and is usually transported into bacterial cells through the PTS. Unlike other sugar-specific EIIs, most mannose transporters have an additional EIID domain and transport various substrates in addition to mannose in the family *Enterobacteriaceae*. Mannose PTS transporters display limited distribution across bacteria and are mostly present in species that are associated with animals^7^. In particular, mannose PTSs are abundant in Gram-positive bacteria living on carbohydrate-rich mucosal surfaces^8^. These characteristics suggest that the mannose transporter may play an important role in the symbiotic or parasitic relationships between animals and bacteria^7,8^. For example, in *Edwardsiella piscicida*, an important fish pathogen, host-derived mannose is sensed by the mannose PTS transporter and transported as mannose 6-phosphate (M6P), where the key virulence regulator EvrA directly binds to elevate virulence^9^. In *Lactobacillus johnsonii*, a health-promoting gut bacterium in humans, deletion of the mannose PTS genes decreases the gut residence time^10^. Moreover, in *V. cholerae*, the addition of free mannose has been shown to inhibit biofilm formation^11^.

*Vibrio cholerae* has 25 conserved PTS components and uses ten types of PTS carbohydrates, including glucose, fructose, mannose, and N-acetylglucosamine^12–15^. The PTS transporter encoded by *VC1826* has been annotated as a putative fructose PTS transporter as it comprises the fructose-family EIIABC domains. However, recent reports suggest that VC1826 is a mannose PTS transporter as the deletion of its EIIA or EIIC domain results in growth defects when mannose is provided as the sole carbon source^12,13^. According to the transcriptome analysis of *V. cholerae*, the transcription level of *VC1826* was significantly higher than that of genes encoding other PTS transporters during infection in rabbits and mice^16^. Moreover, a transposon insertion mutation in *VC1826* decreases virulence in the *Drosophila* infection model^17^, implying that VC1826 may play an important role in animal infection. However, the transcriptional regulatory mechanism of *VC1826* has not been investigated in *V. cholerae*. Therefore, this study aimed to identify the transcriptional regulator responsible for *VC1826* expression and to explore whether it affects *V. cholerae* growth and biofilm formation in response to mannose.

## Results

### VC1826 is a mannose PTS transporter and forms an operon with VC1827

**Fig. 1:**
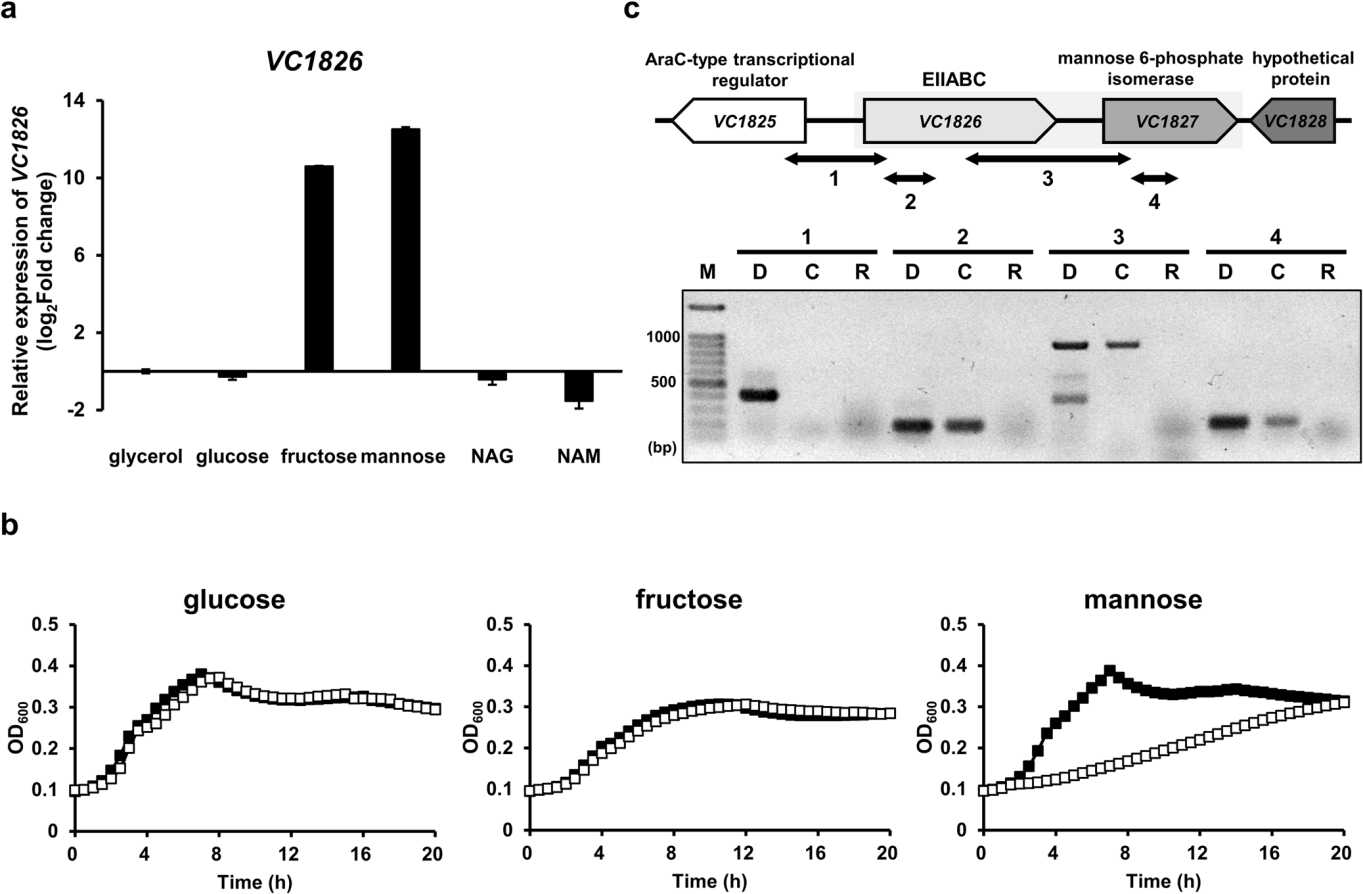
VC1826 is a mannose PTS transporter and forms an operon with VC1827 encoding mannose 6-phosphate isomerase. **a** Relative mRNA expression of *VC1826* in the wild-type *V. cholerae* strain grown on various carbohydrates: NAG, *N*-acetylglucosamine; NAM, *N*-acetylmannosamine. The mRNA expression levels are shown as relative values (log_2_ scale) to that in cells grown on glycerol. The means and standard deviations of three independent measurements are shown. **b** Growth curves of the WT (black squares) and *VC1826* deletion mutant (white squares) in M9 medium supplemented with 0.2% glucose, fructose, and mannose. Growth was measured by recording the absorbance at 600 nm using a multimode microplate reader (TECAN). The means of three independent measurements are shown. **c** Analysis of co-transcription of *VC1826* with *VC1827* using reverse transcription (RT)-PCR. Double-headed arrows with numbers indicate RT-PCR products shown below. RT-PCR products (lanes C) were electrophoresed on an 1% agarose gel with the corresponding PCRs without reverse transcriptase (lanes R); PCR products were amplified using genomic DNA (lanes D) as a template to verify primer specificity. Lane M refers to the DNA size marker (ThermoFisher Scientific).

It was previously shown that, while *V. cholerae* could not grow on any PTS sugars when the EIIC domains of all PTS transporters were deleted, the presence of VC1826 facilitated growth on mannose and fructose. Moreover, a growth defect was observed on mannose, however, not on other PTS sugars, when the EIIA or EIIC domain of VC1826 was deleted in the wild-type (WT) background^12,13^. These results suggest that VC1826 is a PTS transporter that transports mannose and fructose. As the transcription levels of the PTS transporter genes are regulated according to the availability of the substrates^18,19^, we assessed the substrate specificity of VC1826 by determining transcription levels of *VC1826* in the presence of various carbohydrates. Compared with that in the presence of glycerol, a non-PTS sugar, there were slight or no differences observed in the transcription level of *VC1826* in glucose, N-acetylglucosamine (NAG), and N-acetylmannosamine (NAM), whereas it increased by over one thousand-fold in the presence of mannose and fructose (Fig. 1a). Next, to confirm whether VC1826 can transport mannose and fructose, we constructed a strain lacking *VC1826* (Δ*VC1826*) to compare its growth with that of WT (Fig. 1b). As expected, there was no growth difference in the presence of glucose, however, the *VC1826* deletion mutant exhibited severely impaired growth on mannose. Meanwhile, the WT and Δ*VC1826* strains showed no growth difference on fructose. *V. cholerae* has a *fru* operon comprising *fruBKA*, which encodes the fructose-specific PTS that transports fructose (*fruB* encoding the FPr-EIIA and *fruA* encoding the EIIB′BC) and the 1-phosphofructokinase FruK. A previous report showed that a *fruA* deletion mutant showed no difference compared with the WT regarding growth on fructose^20^. To examine whether VC1826 may also transport fructose, we constructed a double deletion mutant (Δ*fruA*Δ*VC1826)* and examined its growth on fructose (Supplementary Fig. 1). While the Δ*fruA*Δ*VC1826* strain carrying the empty vector did not show any growth on fructose, the same strain harboring a plasmid bearing *VC1826* could grow on fructose, indicating that VC1826 also transports fructose (Supplementary Fig. 1).

In *Bacillus subtilis*, M6P isomerase (*manA*) forms an operon with the mannose transporter (*manP*)^21^. *VC1827* in *V. cholerae* encodes a mannose 6-phosphate isomerase that converts M6P to fructose 6-phosphate (F6P). Therefore, we hypothesized that *VC1827* may form an operon with *VC1826*. To test this, we performed reverse transcription (RT)-PCR analysis and confirmed that *VC1827* was transcribed along with *VC1826* (Fig. 1c).

Overall, these results suggest that *VC1826* encodes a mannose PTS transporter capable of transporting mannose and fructose and forms a mannose operon (*man* operon) with *VC1827*. Therefore, we designated *VC1826* as *manP* (for mannose-specific PTS permease) and *VC1827* as *manA* (for M6P isomerase).

### VC1825 is the transcriptional activator of the mannose operon

**Fig. 2:**
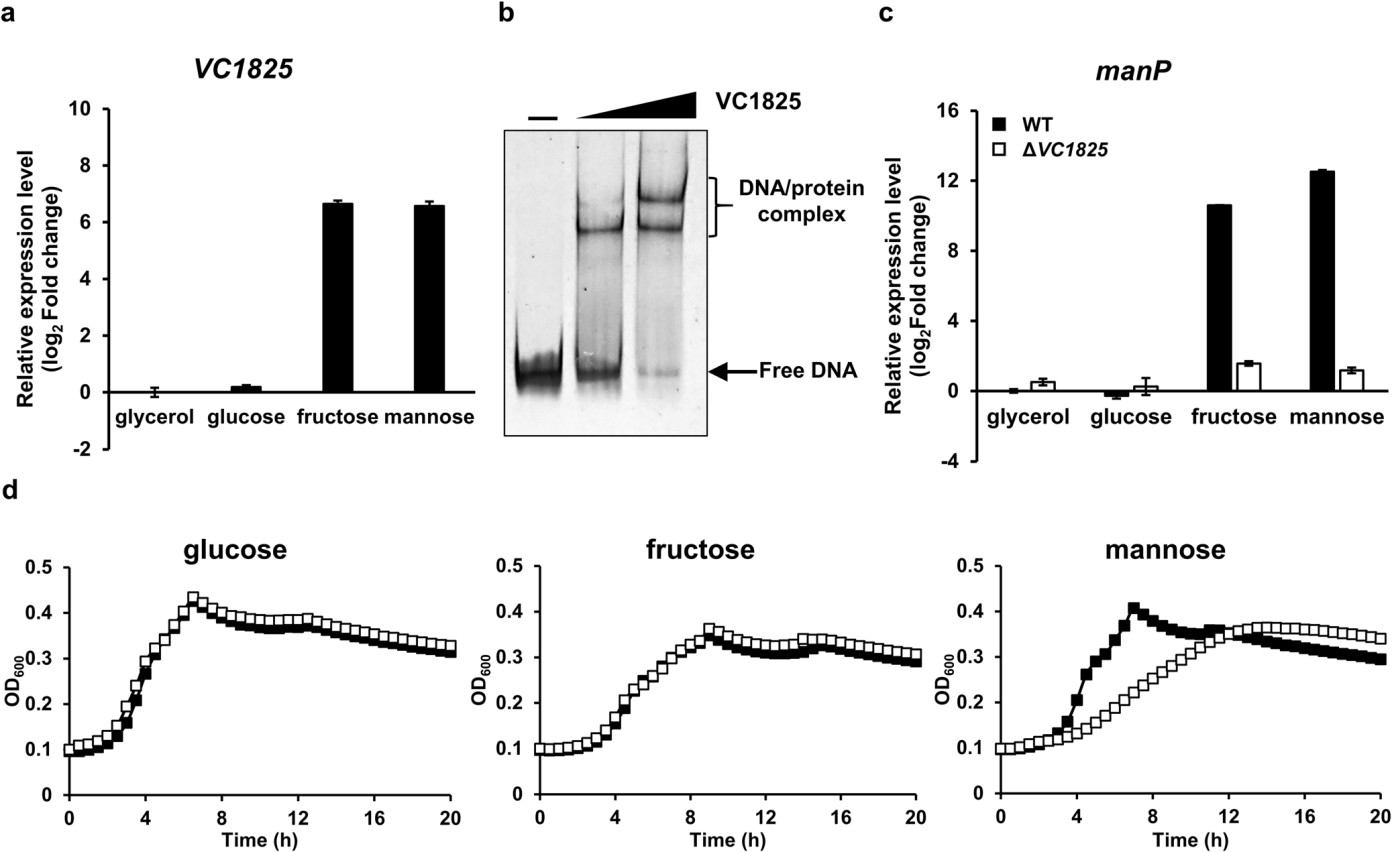
VC1825 activates the transcription of the mannose operon in the presence of mannose and fructose. **a** Relative mRNA expression of *VC1825* in the wild-type *V. cholerae* strain grown on indicated sugars (0.2% each). **b** VC1825 binding to the *manP* promoter was examined using electrophoretic mobility shift assay (EMSA). The 399-bp *manP* probe (60 ng) containing the entire *VC1825–manP* intergenic region was incubated with increasing amounts of VC1825 (0, 60, and 120 ng) and analyzed on a 6% polyacrylamide gel in TBE. **c** Relative mRNA expression of *manP* in the WT and *VC1825* deletion mutant grown on indicated sugars. **d** Growth curves of the WT (black squares) and *VC1825* deletion mutant (white squares) in M9 medium supplemented with indicated sugars. Growth was measured by recording the absorbance at 600 nm using a multimode microplate reader (TECAN). The means of three independent measurements are shown. The mRNA expression levels of *VC1825* and *manP* are shown as relative values (log_2_ scale) to that of the WT strain grown on glycerol in **a**, **c**. The means and standard deviations of three independent measurements are shown in **a**, **c**.

The regulatory mechanism of mannose PTS genes has been well-characterized in *Escherichia coli* and *B. subtilis*^22–25^. However, the transcriptional regulators of these genes in the two bacteria have different characteristics. In the Gram-negative gammaproteobacterium *E. coli*, the *man* operon comprises *manXYZ* (encoding EIIAB, EIIC, and EIID) and is transcriptionally regulated by the global transcriptional regulators Mlc and FruR, which are highly conserved in *V. cholerae*. Conversely, in the Gram-positive bacterium *B. subtilis*, the *man* operon comprises *manPA-yjdF* (encoding EIIBCA mannose transporter, mannose 6-phosphate isomerase, and a hypothetical protein) and is regulated by the specific regulator ManR, which contains a PTS regulation domain, encoded immediately upstream of the *man* operon. By referring to these transcriptional regulators of the *man* operons identified in *E. coli* and *B. subtilis*, we set out to identify the transcriptional regulator of *manP* in *V. cholerae*. To examine whether the transcription of the *man* operon is regulated by Mlc and FruR in the Gram-negative gammaproteobacterium *V. cholerae* as in *E. coli*, an electrophoretic mobility shift assay (EMSA) was performed with a 399-bp probe containing the entire *VC1825–manP* intergenic region (Supplementary Fig. 2). When the probe was incubated with increasing amounts of Mlc or FruR, band shifts were not observed, whereas Mlc bound the *nagE* and *ptsH* promoters and FruR bound the *fruB* promoter. Furthermore, it was recently reported that the deletion of *fruR* did not alter *manP* expression^20^. Hence, we concluded that Mlc and FruR do not regulate gene expression of the *man* operon in *V. cholerae*. Next, we examined whether an uncharacterized AraC-type transcriptional regulator VC1825, located immediately upstream of the *man* operon (Fig. 1c), plays a role in transcription of the *man* operon in *V. cholerae*. Most members of the AraC-type proteins are transcriptional activators involved in the control of several processes related to carbon metabolism, stress response, and pathogenesis^26^. Typically, AraC-type proteins comprise two domains: a non-conserved domain that appears to be involved in effector/signal recognition and dimerization and a conserved domain characterized by significant amino acid sequence homology that extends over 100 residues, including the DNA-binding domain^27^. VC1825 also has an AraC-type protein-specific conserved domain at its C-terminus and forms a dimer (Supplementary Fig. 3). We also confirmed that the expression of *VC1825* was activated in the presence of mannose or fructose in the medium (Fig. 2a). Therefore, we tested whether VC1825 binds the *manP* promoter by EMSA. When the 399-bp probe was incubated with increasing amounts of VC1825, two DNA-protein complexes were formed (Fig. 2b). Thereafter, qRT-PCR experiments were performed to determine whether the expression of the *man* operon was altered following *VC1825* deletion. Results show that the transcriptional induction of *manP* and *manA* mediated by mannose or fructose in the medium was no longer observed in Δ*VC1825* cells (Fig. 2c and Supplementary Fig. 4). Furthermore, the Δ*VC1825* strain showed impaired growth on mannose, similar to the Δ*manP* strain (Fig. 2d). Therefore, we concluded that VC1825 is a transcriptional activator of the *man* operon and designated VC1825 as ManR (for the regulator of the mannose operon). The expression of *manP* and the growth defect present in the Δ*manR* mutant on mannose were restored in the Δ*manR*/pManR strain expressing ManR in trans from the arabinose-inducible *araBAD* promoter (Supplementary Fig. 5a). Notably, even when the expression of ManR was induced in the Δ*manR*/pManR strain, the expression of *manP* increased only in the presence of mannose and fructose (Supplementary Fig. 5b).

### Two ManR-binding sites are present in the *manR–manP* intergenic region

**Fig. 3:**
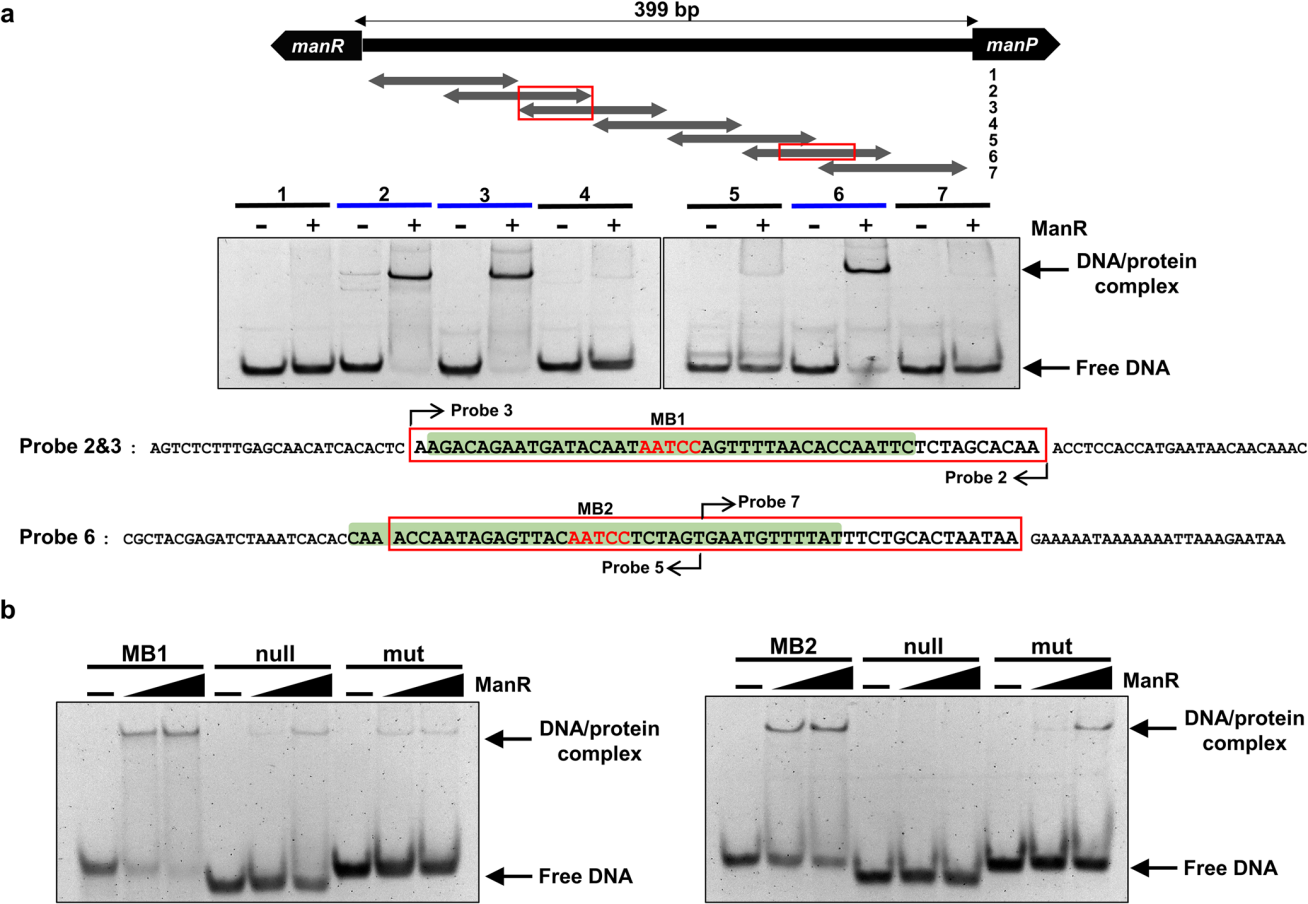
The AATCC sequence in both ManR-binding sites is crucial for DNA binding of ManR. **a** EMSAs were performed using a series of 100-bp probes (represented by double-headed arrows with numbers), each having a 50-bp overlap with the next probe to identify the ManR binding region. Each probe (15 ng) was incubated with (+) or without (−) ManR (120 ng) and analyzed on a 14% polyacrylamide gel in TBE. Blue bars indicate the probes showing band shift, and red boxes indicate the overlapping region of probes 2 and 3 and the central region of probe 6, respectively, the sequences of which are shown below. **b** To determine whether the AATCC sequence is crucial for ManR binding, EMSAs were performed using the 39-bp probes (highlighted in green in **a** encompassing the AATCC sequences and mutated probes where the AATCC sequence was removed (null) or replaced with CACTA (mut). Each probe (8 ng) was incubated with increasing amounts of ManR (0, 60, and 120 ng) and analyzed on a 14% polyacrylamide gel in TBE.

The formation of two DNA-protein complexes in the EMSA experiment (Fig. 2b) suggests that there are two ManR-binding sites (BSs) in the *manR–manP* intergenic region. To define the BSs of ManR more precisely, we constructed a series of 100-bp probes, each with a 50-bp overlap with the next probe, and performed EMSA. Out of the seven probes tested, probes 2, 3, and 6 formed a complex with ManR (Fig. 3a). Therefore, we searched for the consensus sequence in the overlapping region of probes 2 and 3 and in the central region of the probe 6 (boxed in red) and found these probes to share the “AATCC” sequence (in red font). To confirm that this sequence is involved in DNA binding of ManR, we generated 39-bp DNA fragments encompassing the “AATCC” regions (ManR-binding probes, MB1 and MB2, shaded in green in Fig. 3a) and their mutated forms where AATCC is removed (null) or replaced with CACTA (mut). When the DNA fragments were incubated with increasing amounts of ManR, WT MB1 and MB2 each formed one shifted band, whereas mutant fragments showed no or little band shift in EMSA experiments (Fig. 3b). These results suggest that the “AATCC” sequence is important for ManR binding.

### ManR activates the expression of itself and the *man* operon

**Fig. 4:**
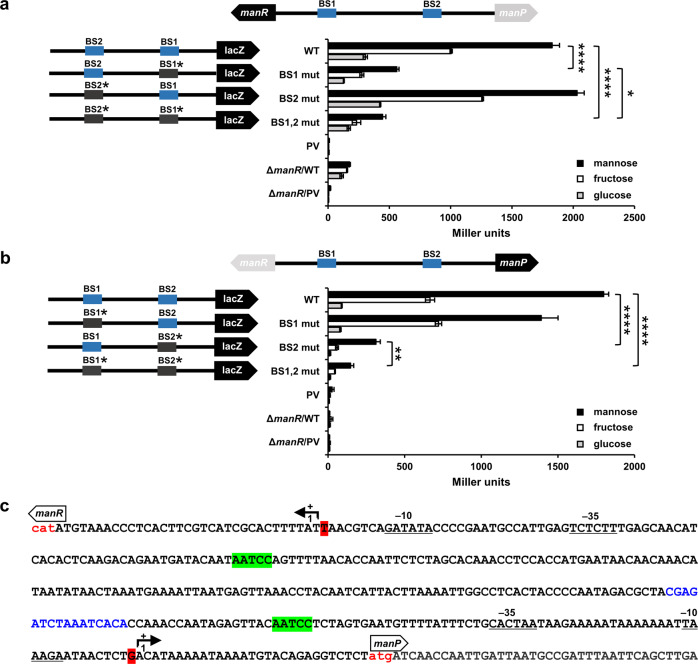
ManR binding to each binding site (BS) activates the expression of itself and the *man* operon. **a**, **b** The effect of ManR binding to each BS on the transcriptional activation of *manR* (**a**) and *manP* (**b**) was measured by the *lacZ* reporter assay using a *V. cholerae* N16961 Δ*lacZ* strain or Δ*lacZ*Δ*manR* strain harboring a plasmid carrying *E. coli lacZ* transcriptionally fused with the wild-type or mutated binding site (mutated site indicated as BS# mut). Schematic representation of plasmids is shown on the left, with mutation site depicted by a gray box and star. Indicated strains were grown on mannose, fructose, or glucose and then lysed to measure the β-galactosidase activity (right panel). The means and standard deviations of three independent measurements are shown. Statistical significance was determined using the Student’s *t*-test (*P < 0.05, **P < 0.005, and ****P < 0.0001). **c** Nucleotide sequence of the *manR–manP* intergenic region. The AATCC sequences of ManR-binding sites are highlighted in bright green. The transcription start sites (TSSs) of *manR* and *manP* are highlighted in red and marked with bent arrows. The −35 and −10 elements of the *manR* and *manP* promoters are underlined, respectively. The initiation codons of *manR* and *manP* are shown in red lower case letters. The putative cyclic AMP (cAMP) receptor protein (CRP) binding site is shown in blue.

We sought to investigate whether the AATCC sequences affect the transcriptional activity of *manR* or *manP* in vivo and how each of the two BSs contributes to the transcriptional activation of these genes. For this, we constructed a set of eight plasmids, each carrying *E. coli lacZ* transcriptionally fused with the *V. cholerae manR* or *manP* promoter (P_*manR*_, P_*manP*_) with the two BSs, either WT or mutated (AATCC to CACTA) and transformed the *V. cholerae* N16961 Δ*lacZ* strain (*manR*^+^) and Δ*lacZ*Δ*manR* strain (*manR*^–^) with one of these plasmids (Fig. 4a, b). We then measured the P_*manR*_ and P_*manP*_ activity in all strains grown either on mannose, fructose, or glucose by measuring the β-galactosidase activity. In the case of the *manR*^+^ strain carrying the WT BSs, cells grown on mannose and fructose exhibited significantly higher P_*manR*_ and P_*manP*_ activities than cells grown on glucose, whereas the *manR*^–^ strain carrying the WT BSs exhibited low β-galactosidase activities regardless of the sugar source. The introduction of mutation at BS1 (BS1 mut) significantly reduced P_*manR*_ activity, while only slightly impacting P_*manP*_ activity. Conversely, the introduction of a mutation at BS2 (BS2 mut) had negligible effects on P_*manR*_ activity, however, significantly decreased P_*manP*_ activity, compared with the strain carrying the WT BSs. As expected, the introduction of mutations at both BS1 and BS2 (BS1,2 mut) resulted in significantly lower activities of both P_*manR*_ and P_*manP*_. These results indicate that the binding of ManR to the AATCC motif in BSs is important for the transcriptional activation of *manR* and the *man* operon. In particular, BS1 is necessary for *manR* expression, and BS2 necessary for *manP* expression; both sites are occupied in the presence of mannose.

The regulatory effect of a transcription factor is usually determined by the location of its BS(s) relative to the transcription start site (TSS)^28^. Activator-binding sites are generally present upstream or next to the −35 core promoter element^28,29^. The TSS of *manR* has been determined using differential RNA sequencing^30^. However, since the TSS of *manP* still remains unknown, we performed a 5′-rapid amplification of cDNA ends (5′-RACE). Consequently, we identified the *manP* TSS located 30 bp upstream of the *manP* initiation codon (Fig. 4c). To further confirm the TSS of *manP*, RT-PCR was performed using RNA isolated from WT N16961 cells grown on mannose or glucose. While the mannose-activated transcript could be detected using the forward primers annealing to sequences downstream of the TSS with more than one thousand-fold activation, regardless of the primer sets used, the transcript was not detected using a forward primer annealing to the sequence upstream of the TSS (Supplementary Fig. 6). Therefore, we concluded that the TSS of *manP* is located 30 bp upstream of the initiation codon of *manP*, and each ManR BS is located upstream of the −35 core promoter element (BS1: −72 to −76 from the *manR* TSS, BS2: −61 to −65 from the *manP* TSS).

### ManR activates transcription of the *man* operon as well as itself by binding to either M6P or F1P

Although ManR was abundantly expressed in the Δ*manR* strain carrying pManR, the expression of *manP* did not increase in the presence of glucose (Supplementary Fig. 5b). In the *manR–manP* intergenic region, there is a BS for cyclic AMP (cAMP) receptor protein (CRP)^31^. CRP is one of the major transcriptional regulators of carbon metabolism and binds DNA in response to cAMP in Gammaproteobacteria^32^. In the absence of glucose, phosphorylated EIIA^Glc^ stimulates adenylyl cyclase, an enzyme that converts ATP to cAMP, which is required for CRP to regulate target genes^33^. Therefore, we assumed that the transcriptional activation of *manP* may require not only ManR but also CRP. To confirm that CRP forms a complex with cAMP and binds to the *manR–manP* intergenic region, EMSA was performed using the 399-bp probe. When we incubated the probe with increasing amounts of CRP, we detected a band shift only in the presence of cAMP (Supplementary Fig. 7a). We then performed qRT-PCR to confirm whether the DNA binding of the cAMP-CRP complex affects the expression of *manP*. The strain with deletion of *crr* gene, encoding EIIA^Glc^, sustains significantly lower cAMP levels than the WT strain^34,35^. When cAMP was exogenously added to the *crr*-deleted strain, *manP* expression was only slightly higher than in the mutant strain incubated in the absence of cAMP, regardless of the type of sugar. However, *manP* expression was significantly increased in the *crr* mutant when incubated in the presence of mannose or fructose, regardless of the addition of cAMP (Supplementary Fig. 7b), indicating that ManR is the major regulator of *manP* expression.

**Fig. 5:**
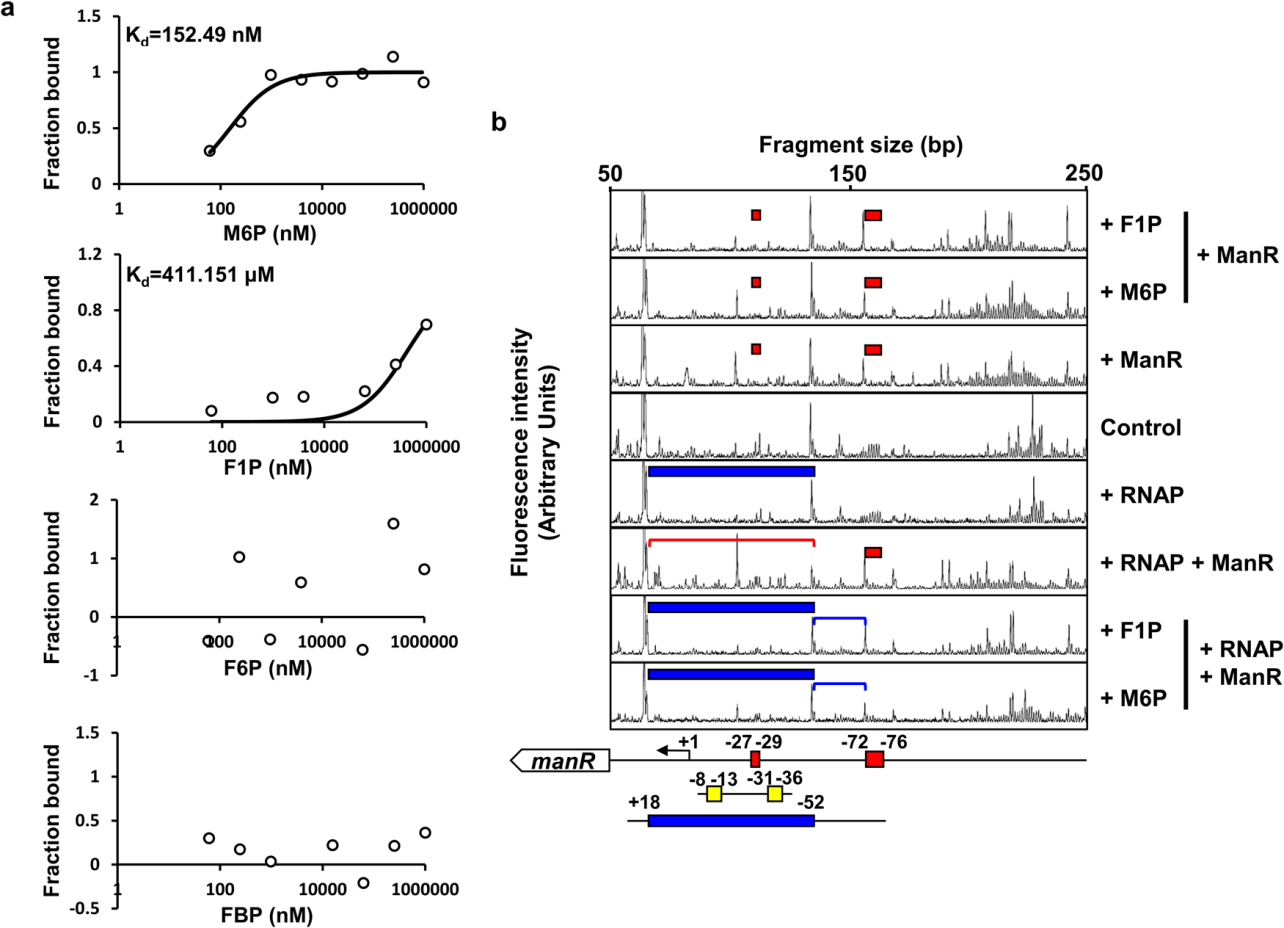
ManR binds to M6P and F1P to facilitate RNAP binding to the promoter. **a** Binding affinities of ManR with mannose 6-phosphate (M6P), fructose 1-phosphate (F1P), fructose 6-phosphate (F6P), and fructose 1-phosphate (F1P), measured using NanoTemper Monolith NT.115^pico^. The dissociation constants (K_d_) of ManR with M6P and F1P were obtained as approximately 152.49 nM and 411.15 μM, respectively. **b** Effect of M6P or F1P on ManR-mediated transcriptional activation of *manR* was assessed by DNase I footprinting assay. A 6-FAM-labeled *manR* probe (200 ng) was incubated with either hybrid RNAP holoenzyme (0.7 μg of *E. coli* core RNAP and 1.4 μg *V. cholerae* σ^70^) or ManR (700 ng) in a different combination in the absence or presence of 2 mM M6P or F1P as indicated. The DNA regions encompassing −52 to +18 and −71 to −53 relative to the *manR* TSS are indicated with red and blue lines, respectively. The DNA regions protected from DNase I digestion by ManR (red bars) and RNAP (blue bars) were then determined. Schematic diagrams of the *manR* promoter region is shown below, and ManR-binding sites, −35 and −10 elements, and RNAP-binding sites are depicted in red, yellow, and blue rectangles, respectively. Nucleotide positions are numbered relative to the *manR* TSS and the bent arrow indicates the *manR* TSS. The fluorescence intensity of the 6-FAM-labeled fragments is shown on the *y*-axis of each electropherogram and fragment sizes were determined by comparison with the internal molecular weight standards.

AraC-type regulators often bind effector molecules and affect transcriptional regulation of target promoters^27^. Both in the WT strain and the Δ*manR* strain carrying pManR, the expression of *manP* did not increase in the absence of mannose or fructose. Therefore, we assumed that ManR requires a co-activator(s) to function as a transcriptional activator. Mannose and fructose are transported as M6P and fructose 1-phosphate (F1P), and subsequently converted to F6P by ManA and fructose 1,6-bisphosphate (FBP) by FruK, respectively. Therefore, we selected M6P, F6P, F1P, and FBP as effector molecule candidates and performed microscale thermophoresis to confirm that these molecules bind to ManR. Interestingly, while ManR did not bind to F6P or FBP, it did bind very tightly to M6P (K_d_ ~ 152 nM), and to a significantly lesser extent, to F1P (K_d_ ~ 411 μM) (Fig. 5a).

Recruitment of RNA polymerase (RNAP) is an important mechanism of transcriptional activation by AraC-type transcriptional activators^36^. To further examine the effects of M6P or F1P on ManR-mediated transcriptional activation of *manR* or the *man* operon, we conducted DNase I footprinting assay with the hybrid RNAP holoenzyme consisting of *E. coli* core enzyme and *V. cholerae* σ^70^ and ManR in the absence or presence of M6P or F1P, using a DNA probe encompassing −166 to +34 relative to the TSS of *manR*^20^. In this promoter, we observed two regions protected by ManR (indicated with red boxes in Fig. 5b), with the AATCC motif in BS1 being the most prominent binding site. The protection of this region by ManR was little affected by the addition of M6P or F1P. As expected from the predicted nucleotide position of the *manR* TSS based on the previous TSS-seq data^30^ (Fig. 4c), RNAP holoenzyme protected the DNA region from −52 to +18 (blue boxes). However, when RNAP was added to the DNase I reaction mixture together with ManR, the protection by RNAP in the region encompassing −52 to +18 was abolished (red line in Fig. 5b), whereas the protection by ManR at the DNA region containing BS1 occurred regardless of the presence of RNAP (red box in Fig. 5b). This result implies that ManR can interfere with RNAP binding to the *manR* promoter in the absence of mannose or fructose, which results in decreased transcription of the *man* operon and *manR* in the presence of glucose (Fig. 4a, b). It is worth to note that, when M6P or F1P was added to the reaction mixture containing the RNAP holoenzyme and ManR, the protection of the region encompassing −52 to +18 became stronger (compare the regions marked with blue box and red line in Fig. 5b) and the region from −72 to −53 was further protected (Blue line) compared to when either of RNAP or ManR was added. It should be noted that the DNA region from −55 to −40 corresponds to the UP element, which is the binding site of the C-terminal domains of the α subunits of RNAP; it further increases the association of RNAP with DNA and thereby stimulates transcription^37^. Therefore, these results suggest that ManR-M6P or ManR-F1P complex facilitates the RNAP binding to the promoter, which result in the transcriptional activation of *manR* and the *man* operon in the presence of mannose or fructose.

### *V. cholerae* ManR regulates biofilm formation and host infection

**Fig. 6:**
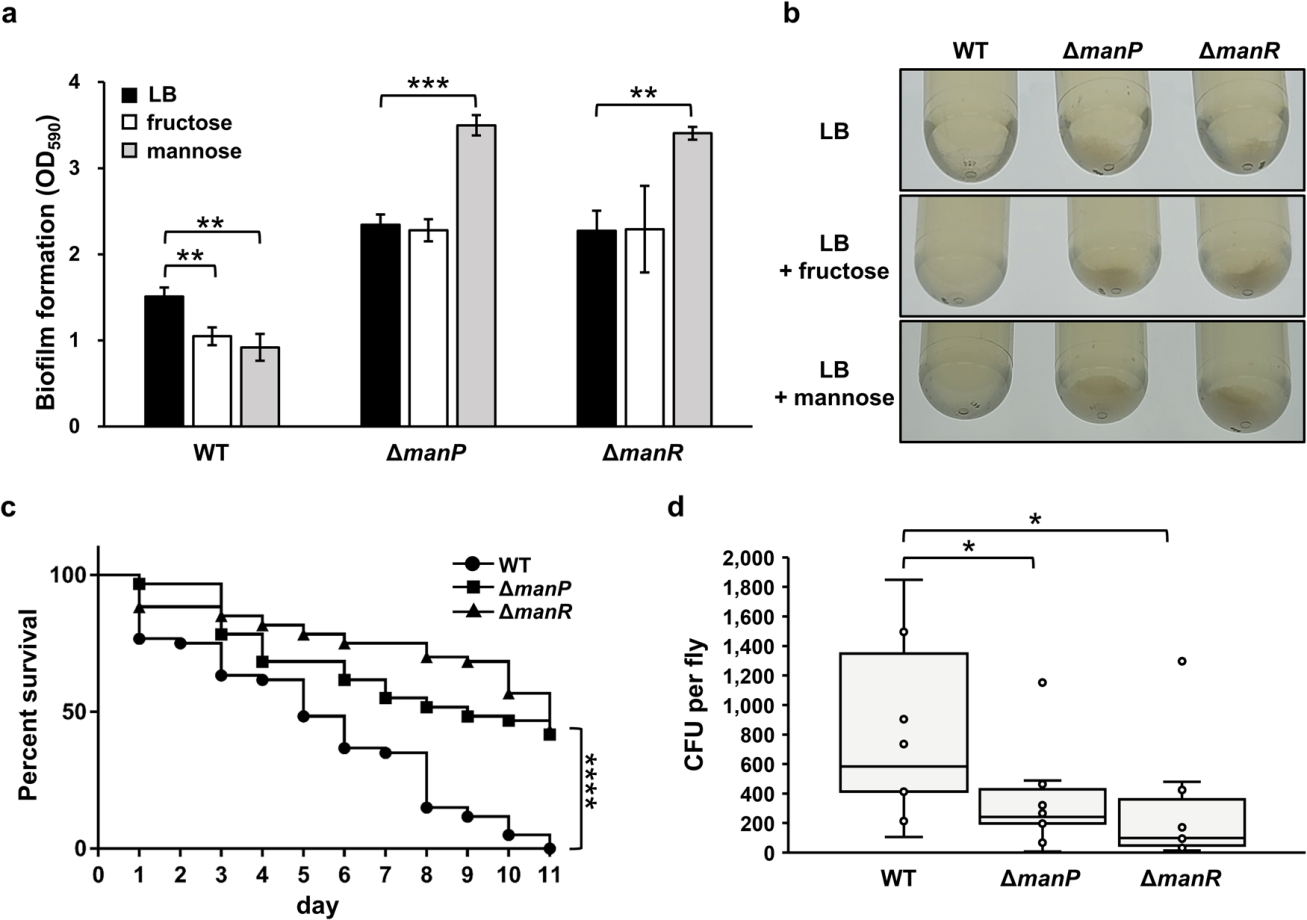
*V. cholerae* ManR regulates biofilm formation, intestinal colonization, and virulence in *Drosophila*. **a** Biofilm formation of WT, Δ*manP,* and Δ*manR V. cholerae* strains measured in LB supplemented with fructose or mannose using the crystal violet staining method following the static growth of *V. cholerae* cells for 24 h. The stained biofilm was determined at 590 nm. The means and standard deviations of the three independent measurements are shown. **b** Macroscopic analysis of autoaggregation of WT, Δ*manP,* and Δ*manR* strains. The indicated strains statically cultured in LB supplemented with fructose or mannose. After 24 h, photographs of the bottom of culture tube were taken. **c** Survival of flies was assessed by infection assays using WT, Δ*manP,* and Δ*manR*. Flies were fed a solution containing approximately 1.6 × 10^10^ cells ml^−1^ of the indicated *V. cholerae* strains. Flies were transferred into new feeding vial and fed a new solution every day, and dead flies were counted. Survival curves were plotted and analyzed by the log-rank test using GraphPad PRISM version 6 software (****P < 0.0001). **d** Intestinal colonization of *V. cholerae* in flies was assessed for a short infection period. Flies were fed a solution containing approximately 1.6 × 10^10^ cells ml^−1^ of the indicated *V. cholerae* strains for 4 h and then fed sterile water for another 3 h for clearing processes. Each fly gut was homogenized in sterile water and spread on a selective medium for *V. cholerae* and colony-forming units (CFUs) were measured. The means and standard deviations of the ten independent measurements are shown. Statistical significance was determined using the Student’s *t*-test (*P < 0.05, **P < 0.005, ***P < 0.0005, and ****P < 0.0001).

In *V. cholerae*, mannose inhibits biofilm formation^11^. Considering that ManP transports mannose and fructose, we performed biofilm formation assays to determine whether the effect of mannose on biofilm formation is influenced by ManP and/or ManR. To this end, we measured the biofilm production of WT, Δ*manP*, and Δ*manR* in the presence of either mannose or fructose in Luria-Bertani (LB) medium (Fig. 6a). The biofilm formation of the WT *V. cholerae* N16961 strain was significantly reduced by the addition of mannose or fructose, compared with in the absence of sugar. However, when either *manP* or *manR* was impaired, biofilm formation was higher than that of WT in LB and the inhibitory effect of mannose and fructose on biofilm formation was completely abolished. Interestingly, biofilm formation was rather increased by mannose in both *manP* and *manR* mutant strains.

Next, to investigate how mannose influences biofilm formation, we compared the levels of autoaggregation, which is an essential step in biofilm formation^38^, among the WT, Δ*manP,* and Δ*manR* strains in the absence and presence of fructose or mannose (Fig. 6b). We statically cultured these strains in LB medium in the presence of mannose or fructose. Cells of the Δ*manP* and Δ*manR* strains were observed to aggregate and settle at the bottom of the cultured tube in LB medium and showed significantly higher autoaggregation in the presence of mannose than in the presence of fructose, whereas WT cells did not aggregate regardless of medium. This result is consistent with the biofilm formation data (Fig. 6a). Thus, we assume that ManR affects biofilm formation and autoaggregation by regulating *manP* expression in response to mannose and, to a lesser extent, to fructose.

The fruit fly *Drosophila melanogaster* is an appropriate model organism for elucidation of host susceptibility to *V. cholerae* due to its simple, yet similar, physiological characteristics and anatomical structure to mammalian infection models^39,40^. It has been reported that the transposon insertion mutation in *manP* decreases *V. cholerae* virulence in the *Drosophila* infection model^17^. To determine whether the regulation of *manP* expression by ManR affects host infection of *V. cholerae*, flies were fed a solution containing approximately 1.6 × 10^10^ cells ml^−1^ of *V. cholerae* strains (WT, Δ*manP*, and Δ*manR*) every day and their viability was monitored. The flies infected with the Δ*manP* or Δ*manR* strain had significantly higher viability than those infected with the WT strain (Fig. 6c), indicating that ManR affects the virulence of *V. cholerae* by regulating *manP* expression in the host. It is also noteworthy that the flies infected with the Δ*manR* strain had slightly higher, although statistically insignificant (log-rank test: *P* ~ 0.496), viability than those infected with the Δ*manP* strain. Considering that in early host infection, *V. cholerae* must spread across the intestinal mucosa rather than forming biofilm (autoaggregation)^41^, we hypothesized that the higher survival rate of flies fed the mutant strains may be due to mutations in *manR* or *manP* causing less efficient spreading to the mucosa, thus, preventing efficient colonization of the gut mucosa. To test our hypothesis, we assessed whether ManR affects the intestinal colonization of *V. cholerae* at the early stage of infection. Flies were fed a solution containing approximately 1.6 × 10^10^ cells ml^−1^ of WT *V. cholerae* or its *manP* or *manR* mutant for 4 h. For clearing processes, flies were fed sterile water for another 3 h and then surface-sterilized, homogenized in sterile water and spread on an agar plate. By measuring colony-forming units (CFUs), we quantified the colonization of *V. cholerae* in vivo. As expected, the CFU of the Δ*manP* or Δ*manR* strain was significantly lower than that of the WT strain (Fig. 6d). In addition, the CFU of the Δ*manR* strain was slightly lower than that of the Δ*manP* strain, although statistically insignificant (Student’s *t*-test: *P* ~ 0.685). These data suggest that ManR enhances intestinal colonization and virulence by suppressing autoaggregation, an essential step in biofilm formation, in response to mannose in the host and this is mainly mediated through the activation of *manP* expression in *V. cholerae*.

### Identification of other ManR regulon genes

**Fig. 7:**
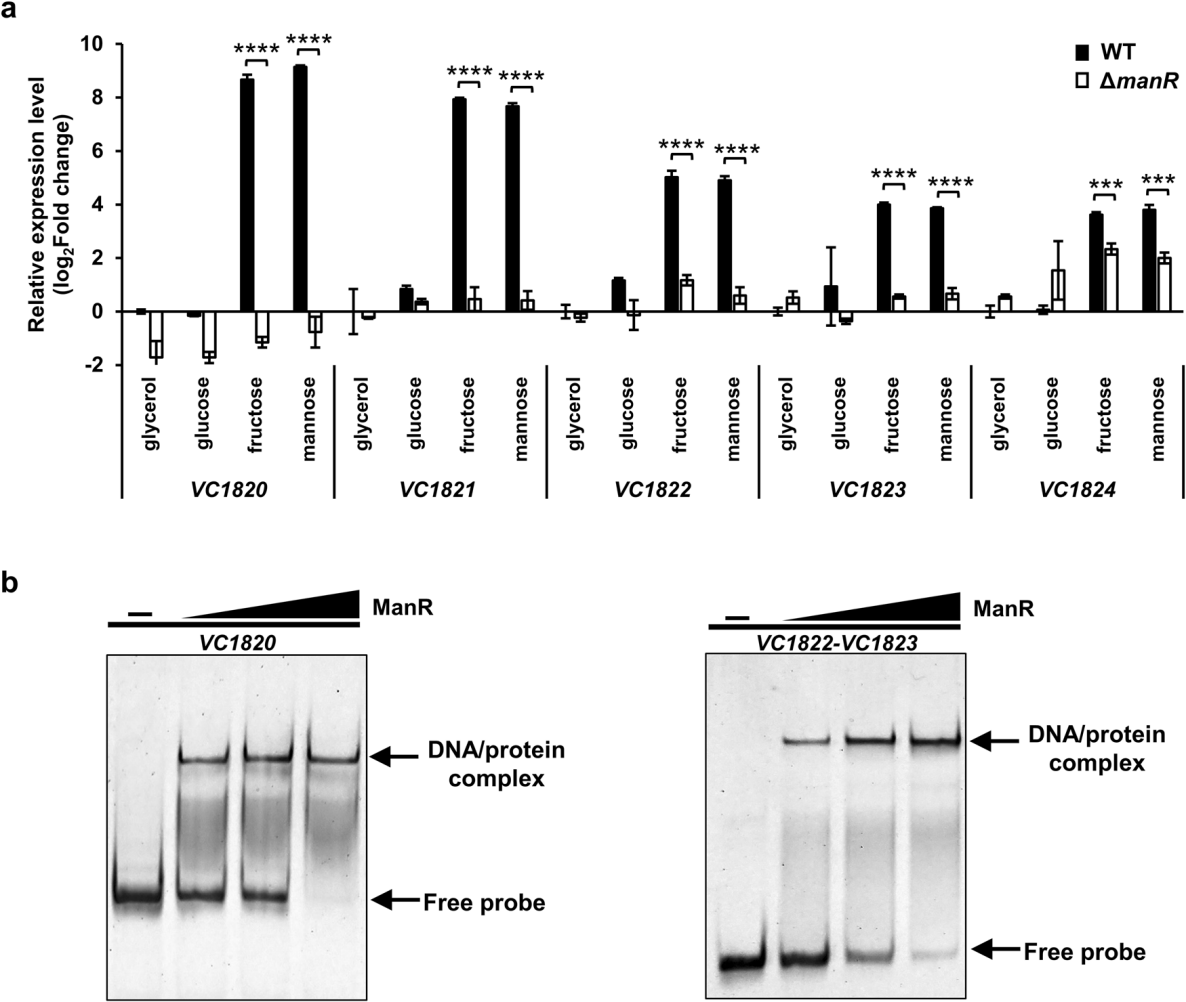
ManR regulates the transcription of *VC1820* to *VC1824* genes as well as the *man* operon and itself. **a** Relative mRNA expression of *VC1820* to *VC1824* genes in the WT and *manR* deletion mutant in the presence of indicated sugars. The mRNA expression levels of indicated genes are shown as relative values (log_2_ scale) to that of the WT strain grown on glycerol. The means and standard deviations of the three independent measurements are shown. Statistical significance was determined using the Student’s *t*-test (***P < 0.0005 and ****P < 0.0001). **b** EMSAs were performed to determine ManR binding to the *VC1820* promoter and *VC1822–VC1823* intergenic region. The probes (20 ng) containing either the entire *VC1820* promoter and the *VC1822–VC1823* intergenic region were incubated with increasing amounts of ManR (0, 30, 60, and 90 ng) and analyzed on a 6% polyacrylamide gel in TBE.

In the *V. cholerae* infection experiment, the Δ*manR* strain showed a slightly greater (albeit statistically non-significant) effect than Δ*manP*. Therefore, we assumed that ManR might regulate other genes as well as *manP*. To identify other possible target genes of ManR, mRNA sequencing was conducted using the RNA samples extracted from the WT *V. cholerae* and *manR* deletion mutant strains, grown on M9 medium supplemented with glucose, fructose, or mannose. By comparing expression levels between WT and the *manR* mutant, we identified genes differentially expressed between the two strains (Supplementary Table 1). Interestingly, the expression of all *VC1820* to *VC1827* genes (Supplementary Fig. 8a) was significantly increased in the WT cells grown on mannose or fructose, but not in the *manR* mutant cells. Genes *VC1820* to *VC1824* encode for PTS components: fructose-family EIIA, fructose-family EIIBC, fructose-family EIIABC, fructose-family EIIB, and nitrogen regulatory EIIA component, respectively. To confirm that ManR regulates the transcription of *VC1820* to *VC1824* genes, qRT-PCR and EMSA were performed. As expected, the expression of these genes increased in the WT strain in the presence of mannose and fructose, however, not in the strain lacking *manR*. In the EMSA experiment, ManR formed one DNA-protein complex with probes containing either the *VC1820* promoter or the *VC1822–VC1823* intergenic region (Fig. 7). In addition, the AATCC sequence was found both in the *VC1820* promoter and *VC1822–VC1823* intergenic region (Supplementary Fig. 8b), suggesting that the *VC1820* to *VC1824* genes also belong to the ManR regulon. Together, our data suggest that the AraC-type transcriptional activator ManR plays a crucial role in intestinal colonization and virulence of *V. cholerae* by regulating PTS-encoding genes in response to mannose and fructose in the host.

## Discussion

The ability to sense and respond to the environment is important for bacterial survival. In many bacteria, the PTS serves as a sensory system that regulates multiple metabolic pathways in response to carbohydrate availability in various environments^4^. Previous reports have shown that the PTS in *V. cholerae* regulates biological processes such as biofilm formation^15,42,43^, colonization of animal hosts^13^, chitin utilization, and natural competence^44^. As the mannose-specific PTS has a limited distribution in bacteria that are associated with animals^7^, elucidating the transcriptional regulation mechanism of the mannose PTS in *V. cholerae* is necessary to understand how it regulates cellular processes in response to the host environment. In this study, ManR, an AraC-family transcriptional regulator, was found to activate the transcription of the mannose operon in the presence of mannose and fructose.

**Fig. 8:**
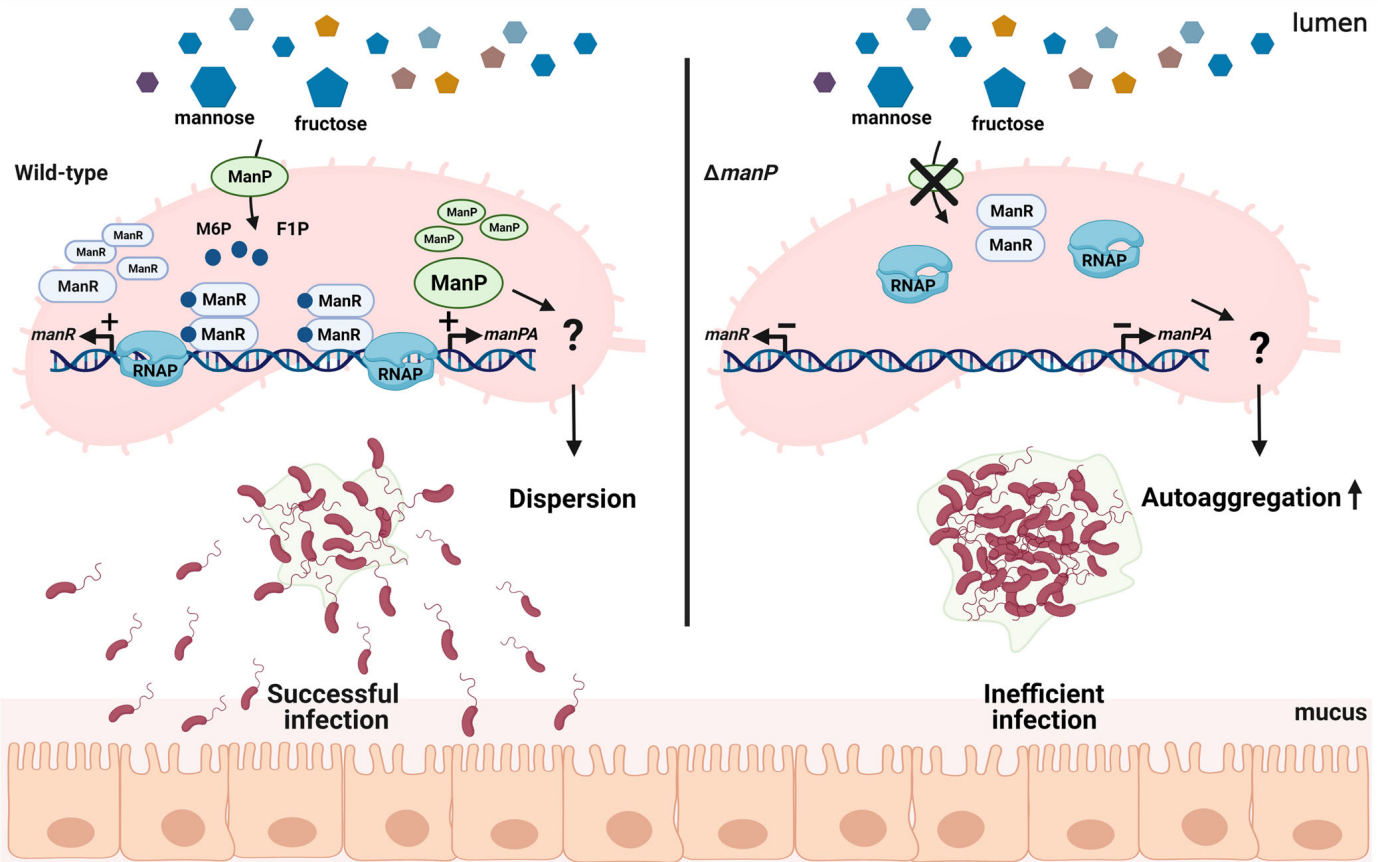
A schematic diagram of the hypothesis for the regulation of biofilm formation by ManR. In the early infection stage, *V. cholerae* senses mannose and fructose in the intestinal lumen and transports them as M6P and F1P, respectively, via ManP. Upon binding to intracellular M6P or F1P, ManR activates the transcription of its regulon genes, including the mannose operon. As *manP* expression increases, bacterial cells will take up more mannose and fructose, constituting a feedforward loop. Because ManP suppresses autoaggregation (through a mechanism yet to be understood, marked with ?), *V. cholerae* cells can disperse more efficiently without biofilm formation, thus can colonize, proliferate, and spread in the host intestine. However, when *manP* is not expressed, cell-cell aggregation is induced, which prevents successful infection. Illustration created using biorender.com.

The AraC-type transcriptional regulator constitutes one of the largest groups of regulatory proteins and is broadly distributed among Gram-negative and -positive bacteria. AraC-type transcriptional regulators are involved in the transcriptional regulation of various cellular processes, in particular, virulence-related gene expression depending on environmental chemicals in several pathogens^45^. *Citrobacter rodentium* RegA, *Providencia stuartii* UreR, and *Streptomyces scabies* TxtT have been reported to control the expression of a set of genes that determine or contribute to pathogenesis in response to bicarbonate, urea, and cellobiose, respectively^45–48^. In *V. cholerae*, the AraC-type transcriptional regulator ToxT activates the transcription of virulence genes, including *ctxAB* and *tcpA*, leading to pathogenesis^49^ in response to various effector molecules, namely, bile as an activator^50^ and bicarbonate as an inhibitor of ToxT activity^51^. Here, we describe an additional example of virulence regulation by an AraC-type transcriptional regulator in response to mannose and fructose in *V. cholerae*. Specifically, ManR binds to M6P or F1P and activates the transcription of mannose-associated genes, leading to reduced biofilm formation (autoaggregation) and increased survival in the host animal intestine (Fig. 8). A transcriptome analysis of *V. cholerae* showed that the transcriptional level of genes coding for fructose-family PTSs in addition to ManP were elevated during host infection^16^, implying that mannose and fructose could be important nutrients for *V. cholerae* in the host.

It was previously reported that ManR is required for the expression of FdgA (facilitated diffusion of glucose, VC1821 coding for a fructose-family EIIBC protein), and acts downstream of FruR^12^. However, our data showed that FruR did not bind to either the *man* operon promoter (Supplementary Fig. 2) or the promoter of *VC1820*, which forms an operon with *VC1821* and codes for an EIIA protein (Supplementary Fig. 9), suggesting that ManR regulates *fdgA* expression independently of FruR. In addition, the *fdgA* expression level was significantly lower on glucose than on mannose or fructose (Fig. 7a), indicating that FdgA may have a mannose or fructose-associated role in addition to facilitating glucose diffusion.

In most pathogenic bacteria, biofilm formation facilitates colonization and persistence in the host by protecting the bacteria from host immune responses and many antimicrobial agents. Moreover, biofilm-associated *V. cholerae* are more acid tolerant than planktonic cells, which aids their transit through the low pH environment of the stomach, ultimately allowing them to reach the distal small intestine, a preferred site for colonization in humans^52^. However, during early infection, *V. cholerae* also needs to leave the biofilm structure to spread to the intestinal mucosa; this process occurs in response to intestinal-localized environmental factors, such as bile^53^. Therefore, during the initial stages of intestinal infection of the host, *V. cholerae* ManR may play a crucial role in regulating biofilm formation and spreading in response to environmental carbohydrates (Fig. 8).

Earlier work by Murthy et al. showed that the addition of free mannose in LB medium inhibits biofilm formation of *V. cholerae*^11^, which is consistent with our data in this study. However, the study by Moorthy and Watnick provided evidence that the presence of mannose in minimal medium can enhance the clumping of cells and allow the monolayer develops into a biofilm^54^. The bacterial biofilm formation process includes several stages (initial attachment, microcolony formation, biofilm maturation, and dispersion) and is regulated by various factors in a complex fashion^55^. Therefore, the influence of mannose on biofilm formation is probably rather complex and dependent on circumstances and additional factors. Further study is needed to determine how ManP regulates autoaggregation.

## Methods

### Bacterial strains, culture conditions, and plasmids

Details of strains, plasmids, and oligonucleotides used in this study are listed in Supplementary Tables 2 and 3. All *V. cholerae* N16961 strains were cultured in Luria-Bertani (LB) medium or M9 minimal medium supplemented with the indicated sugars, and *E. coli* strains were grown in LB medium at 37 °C. The following antibiotics were added if necessary: 100 μg ml^−1^ of ampicillin and 2 μg ml^−1^ of chloramphenicol for *V. cholerae*, and 100 μg ml^−1^ of ampicillin and 20 μg ml^−1^ of chloramphenicol for *E. coli*. All plasmids were constructed using standard PCR-based cloning procedures and verified by DNA sequencing. All plasmids, except pDM4-based plasmids, were directly transformed into *V. cholerae* by electroporation^56^. In-frame deletion mutants of *V. cholerae* were generated by allelic exchange using a pDM4-based plasmid. The *E. coli* SM10 λ*pir* strain carrying pDM4-based plasmids was conjugated to *V. cholerae*, and transconjugants were selected on TCBS agar containing chloramphenicol. The stable transconjugants were spread on LB agar containing 10% sucrose to promote allelic exchange. All transconjugants and mutants were confirmed by PCR^20,57^. To construct the P_*manR*_*::lacZ* and P_*manP*_*::lacZ* transcriptional fusion vectors, the *manR–manP* intergenic region was amplified using PCR with the appropriate primer pairs (Supplementary Table 3). The PCR product was digested with SalI (New England Biolabs, Beverly, MA) and inserted into the corresponding site of the pJK1113-based expression vector carrying promoter-less *E. coli lacZ* (pJK-LacZ)^20^. To replace the nucleotide sequences of the ManR-binding sites in the *manR–manP* intergenic region with the mutated sequence (CACTA), site-directed mutagenesis PCR was performed using the appropriate primer pairs (Supplementary Table 3).

### RNA extraction

Overnight-grown *V. cholerae* N16961, Δ*manP*, and Δ*manR* cultures were diluted 100-fold into fresh LB medium and cultured at 37 °C until OD_600_ reached 0.5. The cell pellets were rinsed twice with M9 medium lacking a carbon source. Each culture was divided into several aliquots and supplemented with 0.2% indicated sugar. These samples were cultured for 30 min at 37 °C^20^. After fixing the cells with adding equal volume of 100% methanol for 1 h at −20 °C, total RNA was isolated using the TaKaRa MiniBEST Universal RNA Extraction Kit (Takara Bio, Shiga, Japan) according to the manufacturer’s instructions.

### qRT-PCR

The total RNA (2500 ng) from each sample was converted into cDNA using the EcoDry Premix (Takara Bio, Shiga, Japan). The 30-fold diluted cDNA was subjected to real-time PCR amplification using a FAST SYBR green master mix kit (Life Technologies, Carlsbad, CA) with specific primers in a CFX96 Real-Time System (Bio-Rad, Hercules, CA)^20^.

### Measurement of bacterial growth in sugar-supplemented medium

The *V. cholerae* N16961, Δ*manP,* and Δ*manR* strains were grown in LB medium and resuspended in M9 medium. The cells were inoculated into a 96-well plate containing M9 medium supplemented with 0.2% glucose, fructose, or mannose and cultured at 37 °C. The optical density of all cultures was measured at 600 nm using a multimode microplate reader (Spark 10 M multimode microplate reader, TECAN, Mannedorf, Switzerland).

### RT-PCR

To determine the transcriptional unit and TSS of *VC1826* (*manP*), RT-PCR analysis was conducted. Total RNA was extracted from *V. cholerae* N16961 cells using the TaKaRa MiniBEST Universal RNA Extraction Kit (Takara Bio, Shiga, Japan), and converted to cDNA using the EcoDry Premix (Takara Bio, Shiga, Japan). RNA, genomic DNA, and cDNA were used as templates to verify the transcription units using the appropriate primers (Supplementary Table 3)^58,59^.

### Purification of proteins

The overexpression of proteins was induced in *E. coli* Rosetta (DE3) /pLysSRARE (Novagen, USA) by adding 1 mM isopropyl β-D-thiogalactoside. For untagged proteins, harvested cells were resuspended in buffer A (50 mM Tris-HCl [pH 8.0], 10 mM DTT, 10 mM EDTA, and 10% glycerol) containing 50 mM NaCl and disrupted by ultrasonication. After centrifugation at 15,000 × *g* at 4 °C for 15 min to remove cell debris, the supernatant was filtered with a Hi-Trap Heparin HP affinity column (GE Healthcare Life Sciences, USA). Protein elution was performed using a 20-column volume 0.05–1 M NaCl gradient in buffer A at a flow rate of 2 ml min^−1^. His-tagged proteins were purified using TALON metal-affinity resin (Takara Bio, Shiga, Japan) according to the manufacturer’s instructions. After elution with 200 mM imidazole, the fractions containing His-tagged proteins were pooled. To increase the purity of proteins and remove imidazole, the pool was chromatographed on a Hiload 16/60 Superdex 200 pg column (GE Healthcare Life Sciences, USA) equilibrated with purification buffer (50 mM Tris-HCl [pH 8.0], 5 mM β-mercaptoethanol, 500 mM NaCl, and 10% glycerol)^60^. Proteins were concentrated using Amicon Ultracel-3K centrifugal filters (Merck Millipore, Burlington, MA).

### EMSA

EMSA was performed without using radioactivity^20^. A 399-bp *manP* probe or 100-bp probes were amplified by PCR using the *V. cholerae* N16961 chromosome as a template with appropriate primers (Supplementary Table 3). A 39-bp probe containing a mutated ManR-binding site was constructed by annealing with primers. The probes were mixed with transcription factors in buffer (10 mM Tris-HCl [pH 8.0], 5% glycerol, 0.1 mM EDTA, and 1 mM DTT), and 200 μg ml^−1^ bovine serum albumin (BSA) as non-specific protein competitor. Each sample was incubated at 37 °C for 10 min and then analyzed on a 6% or 14% polyacrylamide gel (acrylamide/bisacrylamide ratio of 29:1) in TBE (89 mM Tris, 89 mM boric acid, and 20 mM EDTA) followed by staining with Green Star nucleic acid staining solution (Bioneer, Daejeon, South Korea). DNA bands were visualized using DUALED Blue/White Transilluminator (Bioneer, Daejeon, South Korea)

### β-Galactosidase assay

A *V. cholerae* N16961 Δ*lacZ* strain or Δ*lacZ*Δ*manR* strain was transformed with the plasmid carrying *E. coli lacZ* fused with the WT or mutated BSs of ManR and grown on mannose to measure the β-galactosidase activities^20,61^. Cultured cells (80 μl) were 10-fold diluted in Z-buffer (60 mM Na_2_HPO_4_, 40 mM NaH_2_PO_4_, 10 mM KCl, 1 mM MgSO_4_, and 40 mM β-mercaptoethanol) and lysed with 20 μl of 0.1% SDS and 40 μl of chloroform at 37 °C for 10 min. The β-galactosidase activity was then measured as described by Miller^62^.

### 5′-RACE

The TSS of *manP* was determined using the SMARTer RACE 5′/3′ Kit (Clontech Lab, Palo Alto, CA) following the manufacturer’s instructions. The cDNA was synthesized using the appropriate primers (Supplementary Table 3) and a poly(C) tail was added to the 3′-end of the cDNA. The 5′-RACE product was amplified by PCR from the poly(C)-tailed cDNA using the Universal Primer Short included in the Kit and the primer specific for the *manP*. The TSS of the target gene was identified by sequencing^58^.

### Microscale thermophoresis analysis

The binding affinities of ManR with several metabolites were measured using a Monolith NT.115^pico^ instrument (Nano Temper technologies, Munich, Germany)^42^. Purified ManR with a C-terminal His tag (ManR-His) was labeled with NT-647 using a Monolith protein-labeling kit and used at a concentration of 2 nM. All metabolites were titrated in 1:3 serial dilutions in MST-binding buffer (50 mM HEPES-NaOH [pH 8.0], 500 mM NaCl, 10% glycerol, 1 mM Tris(2-carboxyethyl)phosphine hydrochloride [TCEP], 0.5 mg ml^−1^ BSA, and 0.05% [v/v] Tween 20), with the highest concentration of each metabolite at 1 mM. The measurements were performed at 10% LED power and Low MST power at 23 °C. Fructose 1-phosphate (F1P) was purchased from Santa Cruz Biotechnology (Santa Cruz, CA) and other metabolites from Sigma-Aldrich (St. Louis, MO), unless otherwise specified.

### DNase I footprinting

DNase I footprinting experiments were performed as described in a previous study with some modifications^20^. The 6-carboxyfluorescein (6’FAM)-labeled *manR* probe encompassing −166 to +34 bp relative to the *manR* TSS was prepared by PCR (Supplementary Table 3). The purified PCR product was incubated with the indicated amounts of proteins and metabolites at 37 °C for 10 min prior to digestion with 0.04 U DNase I (New England Biolabs, Beverley, MA, USA, # M0303S) for 1 min. The cleavage reaction was stopped by adding the same volume of stop solution (200 mM NaCl, 30 mM EDTA, and 1% SDS) followed by phenol extraction and EtOH precipitation. DNase I digestion reactions were analyzed by capillary electrophoresis in an ABI 3730xl DNA Analyzer (Applied Biosystems, Foster City, CA) with Peak Scanner software v1.0 (Applied Biosystems, Foster City, CA).

### Biofilm formation analysis

A microtiter plate assay was used to evaluate biofilm formation^63,64^. Overnight-grown cells were inoculated at 1:100 dilution in LB medium in the absence or presence of 0.2% sugar and incubated under static conditions in sterile 96-well polystyrene microtiter plates (SPL Lifesciences, Gyeonggi-do, South Korea) at 37 °C for 24 h. After planktonic cells were washed away with phosphate-buffered saline (PBS), the remaining biofilm-associated cells were stained with 0.1% crystal violet (CV) for 15 min. After rinsing with PBS three times and air drying, the CV-stained biofilm was solubilized with acetone:ethanol (1:4) and measured at 590 nm.

### Autoaggregation assay

*V. cholerae* strains were shaken overnight in LB at 37 °C, and 3 ml of each culture was transferred into a new culture tube. After mannose or fructose was added to a final concentration of 0.2%, the culture tube was left to stand at 37 °C for 24 h, and then a photograph was taken.

### Fly survival assay

*V. cholerae* strains were harvested during the late exponential phase by centrifugation. The bacterial pellets were washed with PBS and suspended in 5% sucrose solution. Adult male flies (5–6-day-old *D. melanogaster w*^*1118*^) were fed a solution comprising 5% sucrose and approximately 1.6 × 10^10^ cells ml^−1^ of *V. cholerae* strains. Flies were transferred into new feeding vial and fed a fresh solution containing *V. cholerae* strains every day, and dead flies were counted.

### Quantification of *V. cholerae* colonization in the fly intestine

To study the differences between WT *V. cholerae* and its mutant strains in abilities to colonize fly intestines, 5–6-day-old female *w*^*1118*^ flies were fed a solution comprising 5% sucrose and approximately 1.6 × 10^10^ cells ml^−1^ of *V. cholerae* strains for 4 h. Flies were transferred into new vials and fed sterile water for clearing processes. After feeding water for 3 h, each fly was immersed in 70% ethanol for 3 min and then dissected. Each gut was homogenized in sterile water and spread on LB plates containing 10 μg ml^−1^ streptomycin to determine the CFUs of *V. cholerae*.

### mRNA sequencing and RNA-seq analysis

After the RNA of WT *V. cholerae* or Δ*manR* was extracted by the RNA extraction method described in this paper, RNA sample preparation, sequencing and data processing were performed^59^. Total RNA (1 μg) was processed to remove the ribosomal RNA using NEBNext rRNA Depletion Kit (Bacteria) (#E7850, New England Biolabs, Beverly, MA), and the sequencing libraries for RNA-seq were constructed using the NEBNext Ultra II RNA Library Prep Kit for Illumina (#E7770, New England Biolabs, Beverly, MA). Sequencing was carried out using NextSeq 500 instrument (Illumina, San Diego, CA) according to the manufacturer’s instructions. The sequencing adapter removal and quality-based trimming on raw data was performed by Trimmomatic v. 0.36^65^ with TruSeq adapter sequences. Cleaned reads were mapped to reference genome of *V. cholerae* O1 biovar El Tor str. N16961 (GCA_003063785.1) using bowtie2 v.2.3.5^66^ with the ‘--no-spliced-alignment’ parameter. FeatureCounts^67^ was used for counting reads that mapped to each CDS. Retrieved counts were normalized as transcripts per million (TPM).

### Reporting summary

Further information on research design is available in the Nature Research Reporting Summary linked to this article.

## Supplementary information

Supplementary Information

Reporting Summary

## Data Availability

The mRNA sequencing data of *V. cholerae* N16961 and *manR* deletion mutant is available from the Gene Expression Omnibus database under the accession number GSE192900.
