# Supplementary material for: A mannose-sensing AraC-type transcriptional activator regulates cell–cell aggregation of Vibrio cholerae
Source: NPJ Biofilms Microbiomes. 2022 Aug 20;8:65. doi: 10.1038/s41522-022-00331-x (PMC9392796; doi:10.1038/s41522-022-00331-x)
Supplement: Supplementary file 1 — Supplementary Information [file 41522_2022_331_MOESM1_ESM.pdf]

Supplementary Information

A mannose-sensing AraC-type transcriptional activator regulates cell-cell aggregation of *Vibrio cholerae*

Hye-Young Lee[1], Chang-Kyu Yoon[2], Yong-Joon Cho[2], Jin-Woo Lee[3], Kyung-Ah Lee[3], Won-Jae Lee[3], Yeong-Jae Seok[1,2*]

Affiliations:

[1]*Department of Biophysics and Chemical Biology, Seoul National University, Seoul 08826, Republic of Korea*

[2]*School of Biological Sciences and Institute of Microbiology, Seoul National University, Seoul 08826, Republic of Korea*

[3]*School of Biological Sciences, Seoul National University, Seoul 08826, Republic of Korea*

Corresponding Author: *E-mail yjseok@snu.ac.kr; Tel. +82 2 880 4414; Fax +82 2 871 1993

Contents

- Supplementary Figures 1-9

- Supplementary Tables 1-3

- Supplementary References

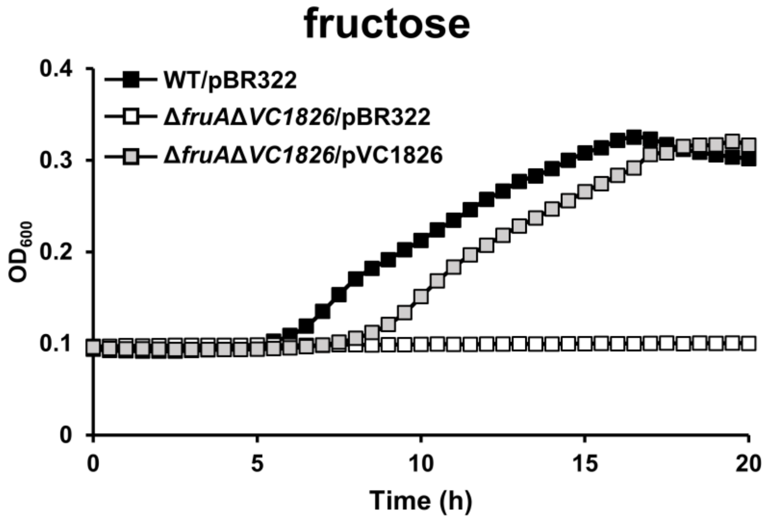

Supplementary Figure 1. VC1826 can transport fructose

The indicated *V. cholerae* strains were cultured in M9 medium supplemented with 0.2% fructose and growth was measured by recording the absorbance at 600 nm using a multimode microplate reader (TECAN). The means of three independent measurements are shown.

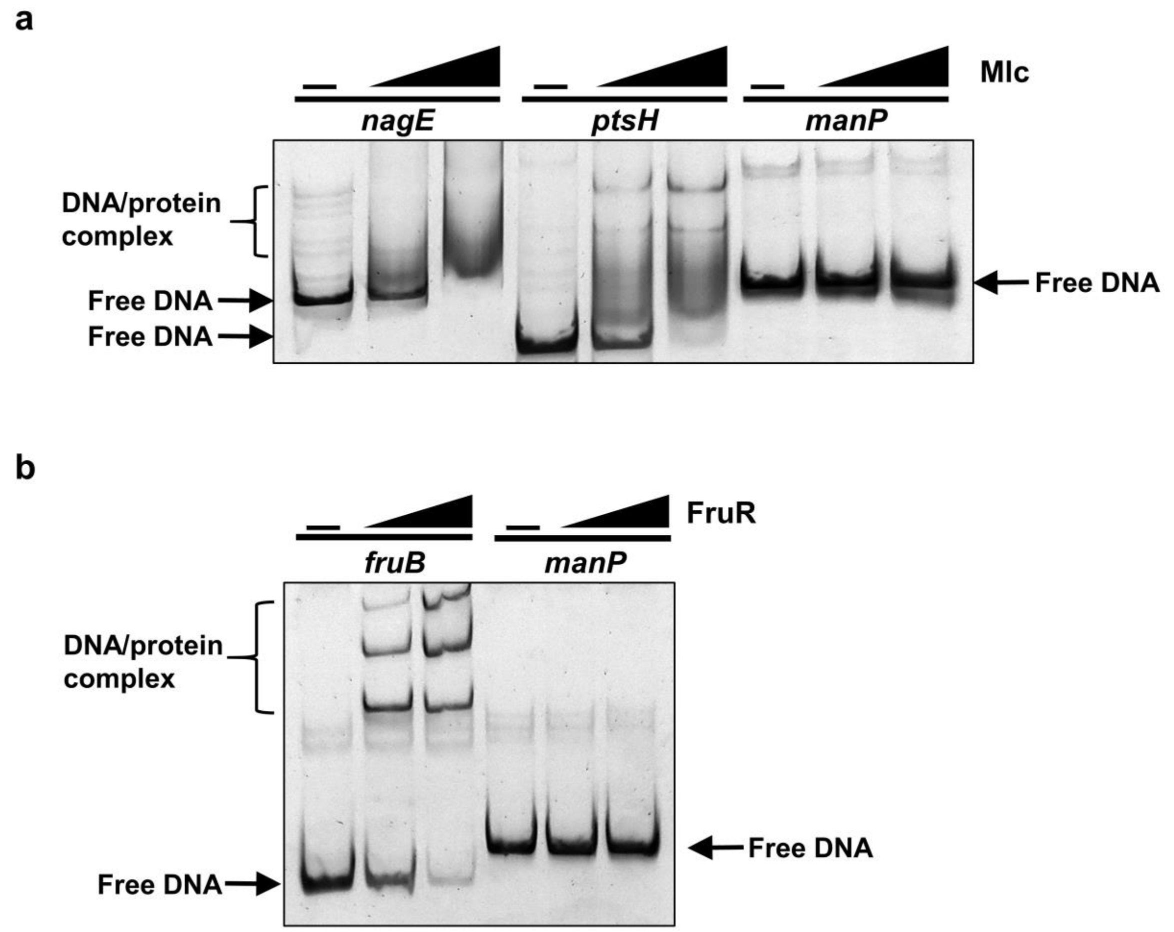

Supplementary Figure 2. Mlc and FruR do not bind to the *VC1825-manP* intergenic region

a Binding of Mlc to the *manP* promoter was tested in comparison with promoter DNAs of two Mlc-regulon genes, *nagE* and *ptsH*, as positive controls. **b** Binding of FruR to the *manP* promoter tested in comparison with the *fruB* promoter DNA as a positive control. Binding of Mlc (0, 60, and 120 ng) and FruR (0, 120, and 240 ng) to the DNA probes (60 ng each) was examined using electrophoretic mobility shift assays on 6% polyacrylamide gels in TBE.

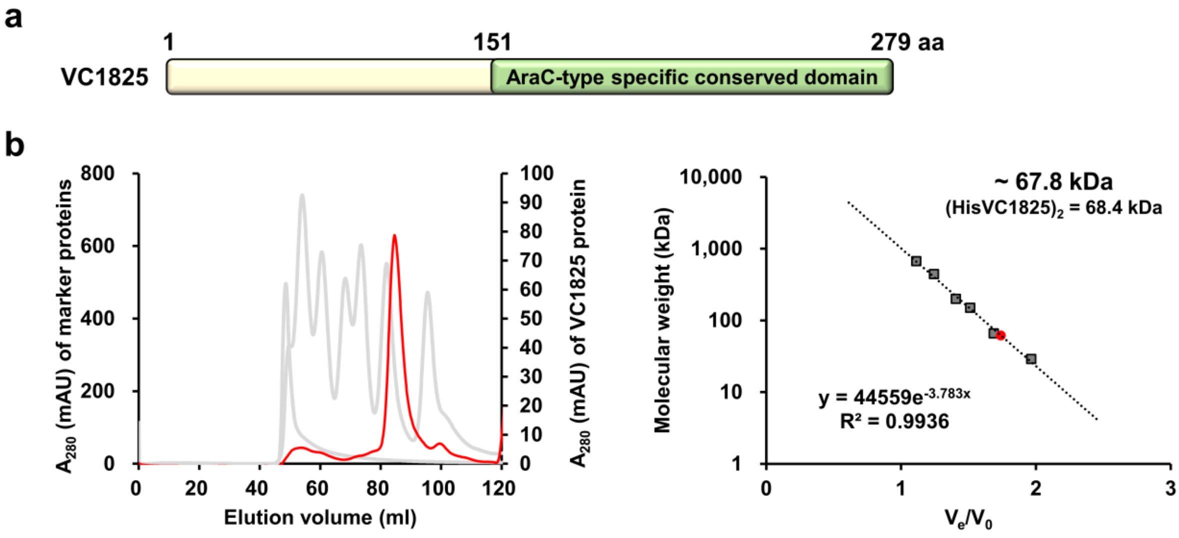

Supplementary Figure 3. The AraC-type transcriptional regulator VC1825 forms a homodimer

a Schematic presentation of the VC1825 polypeptide. **b** Determination of VC1825 molecular weight (MW) by gel filtration chromatography. Samples were applied to a HiLoad 16/600 Superdex 200 prep grade column (GE Healthcare Life Sciences) in a buffer containing 25 mM HEPES-NaOH (pH 7.6), 500 mM NaCl, and 5 mM β-mercaptoethanol, and protein elution was monitored at 280 nm. The column was calibrated using MW markers (Sigma-Aldrich) and blue dextran (gray curve), and the VC1825 elution profile is shown by the red curve (left panel). The void volume (V_0) is the elution volume of blue dextran (~2,000 kDa) and Ve is the elution volume of each protein. From the plot of MW versus the Ve/V_0 value, the MW of VC1825 was estimated from the standard curve to be 67.8 kDa (red dot), supporting its existence as a homodimer (monomer ~34.2 kDa, right panel). MW markers: thyroglobulin (669 kDa), apoferritin (443 kDa), β-amylase (200 kDa), alcohol dehydrogenase (150 kDa), albumin (66 kDa), and carbonic anhydrase (29 kDa).

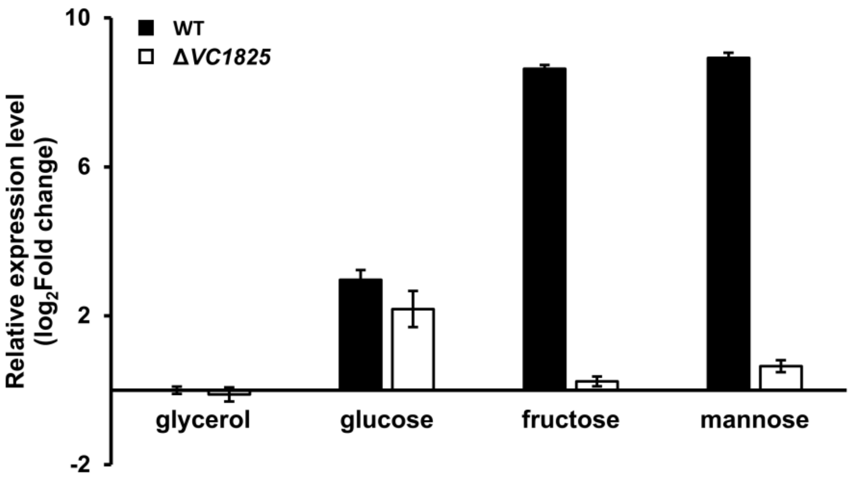

Supplementary Figure 4. The *manA* expression is regulated by VC1825

Transcript levels of *manA* measured in the WT and *VC1825* deletion mutant grown in the presence of indicated sugars (0.2% each) are presented as relative values to that of the WT strain grown on glycerol. The means and standard deviations of three measurements are shown.

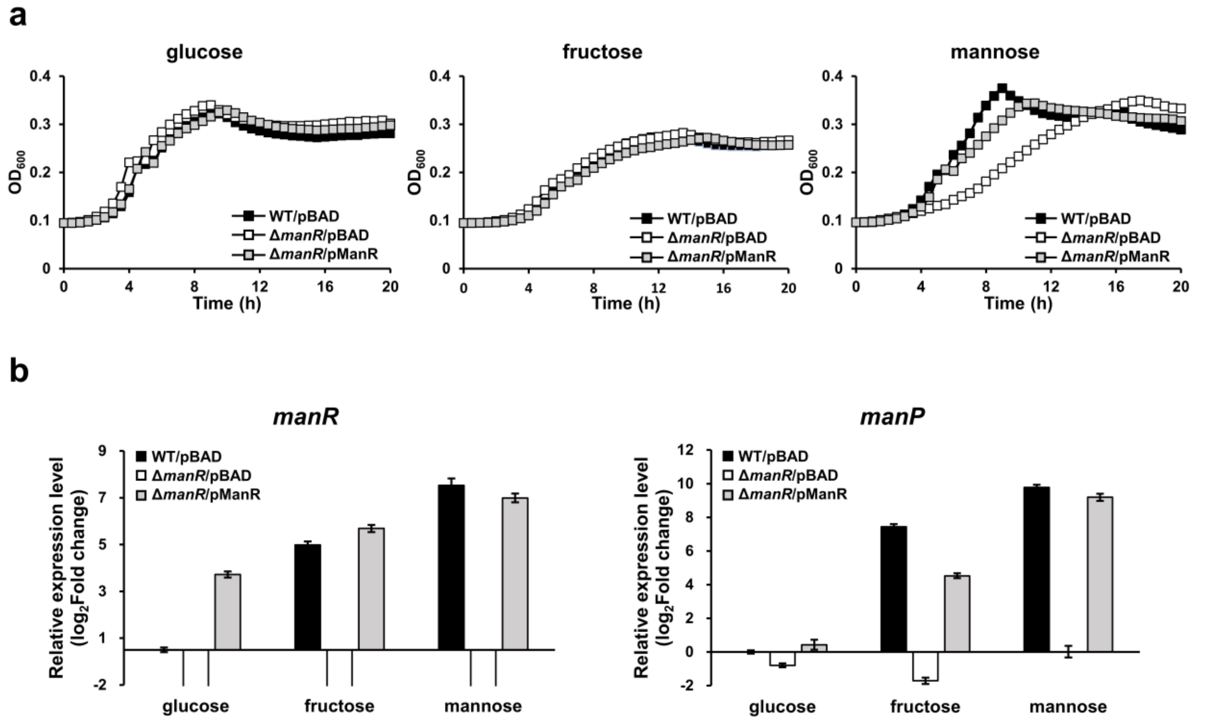

Supplementary Figure 5. *manP* expression and growth on mannose are restored in the Δ*manR* mutant when ManR is expressed in trans

a Growth curves of the indicated *V. cholerae* strains were monitored in M9 medium supplemented with 0.2% glucose, fructose, or mannose as the sole carbon source at 37 °C using a multimode microplate reader (TECAN). The means of three measurements are shown. **b** Relative expression of *manR* and *manP* in cells grown in M9 medium supplemented with 0.2% glucose, fructose, or mannose. The means and standard deviations of three measurements are shown.

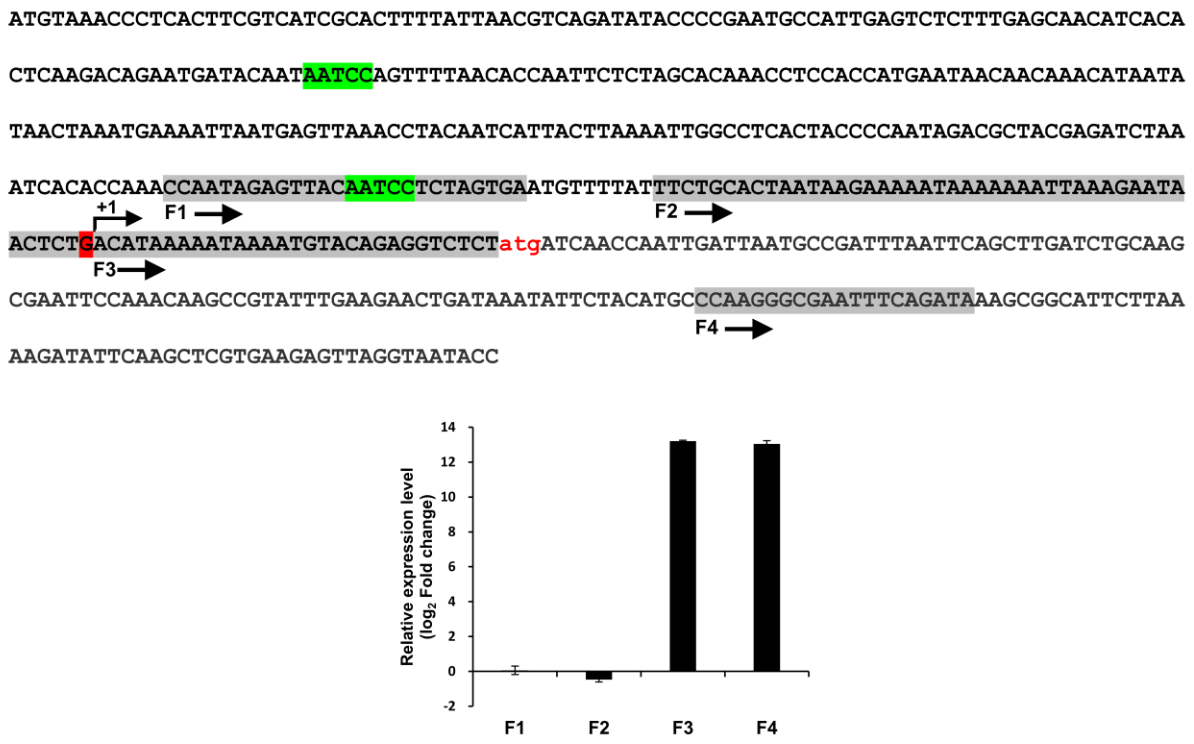

Supplementary Figure 6. Confirmation of the *manP* transcription start site (TSS) by RT-PCR

To confirm the *manP* TSS determined by 5′-RACE, RT-PCR experiments were conducted using RNA isolated from cells grown on mannose or glucose. The TSS and initiation codon of *manP* are shaded in red with a bent arrow and marked in red lower case letters, respectively; the ManR-binding sites are highlighted in bright green. Each forward primer contains the nucleotide sequence highlighted in gray and is indicated by an arrow with a primer number under the nucleotide. RT-PCR was performed using a common reverse primer (TSS-R in Table S3) located in the *manP*-encoding region. The amount of each RT-PCR product using RNA isolated from cells grown on mannose as a template is expressed as a $\log_2$ value compared with that using RNA from cells grown on glucose as a template (bottom panel).

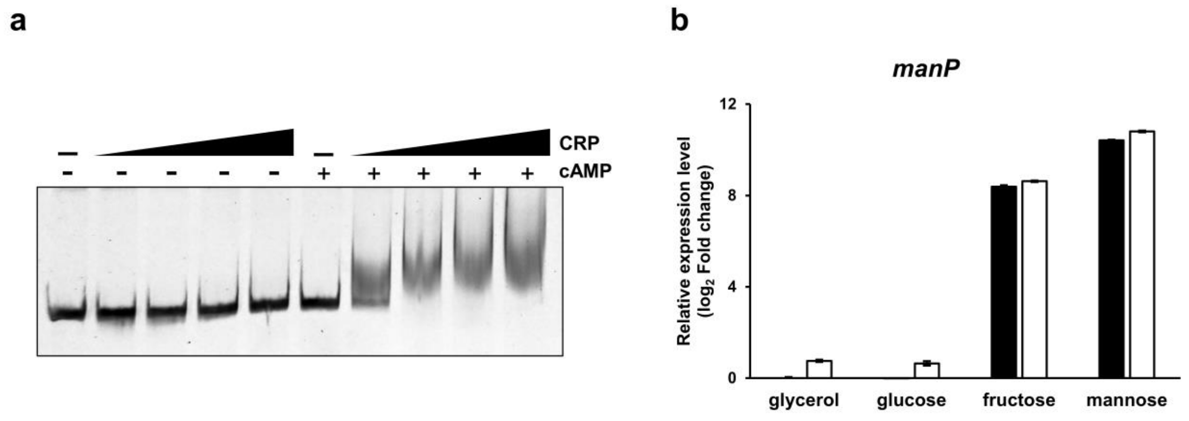

Supplementary Figure 7. The cAMP-CRP complex slightly activates *manP* expression

a EMSA was performed using the 399-bp probe to determine cAMP-CRP binding to the *manR–manP* intergenic region. The probe (40 ng) was incubated with increasing amounts of CRP (0, 100, 200, 300, and 400 ng) in the absence (–) or presence (+) of cAMP (0.2 mM) and analyzed on a 6% polyacrylamide gel in TBE. **b** The mRNA expression levels of *manP* were measured in the *crr* mutant grown in M9 medium supplemented with indicated sugars in the absence (black bars) and presence (white bars) of cAMP (2 mM) and presented as relative values (log_2 scale) to that in the *crr* mutant grown on glycerol in the absence of cAMP. The means and standard deviations of three independent measurements are shown.

a

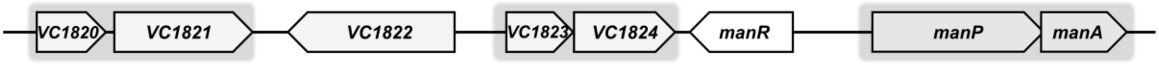

b

>*VC1820* promoter

VC1819

TCGGTTTCTTCtaaAAGCTTAATATTCAGTTAATACCCCAAACCCAGCCTCGTTTCAGCGAAGCTGGGTTTTTCATTTT

GGATCCATTCCAATGTTTTTACGAATTGGCGTTGTAGGGAAACAGCCTGTTAGTTAATCCCGATGAGCATAGATAAACG

ATGTGATTGAGGTGAACGAACGTAGCCAACACCGCTGCCACTTCAAGAAGGAAAGATATATAACGATGAATATCACACT

TAATCATGTGTGGTACAAATCTATAGTGAGTGAATTATTTTTGCTATTTCAGTAAAACTCGACATCCATTAATTTTAGC

VC1820

TCATCAGTTAAATACTTTATGAGCCAATAAatgATCACCGAGCT

>*VC1822–VC1823* intergenic region

VC1823

GCAACAATTTTcatGGAAATCCCTCATTGTTATTTTTGCGGCGATAGTAACATAGCGCTTGGCAAATACTCCGTTTTCG

GCCAAGACCCAGTTCACAGTTCAAATCGAATGTTACATTAATCCAGTTATTGCATCTTTTGTCCTGTGTCTTGAAAGAG

VC1822

TATCGCTCGTTACTCTAGCGAGGCGCATCGGTATGGCAATGCCAATTCAAACAACAGTAAGGTAAAACAATTgtgGATA

TTACCCA

Supplementary Figure 8. The ManR-binding sequence AATCC presents in other ManR target promoters

a Graphical depiction of the *VC1820* to *VC1827* genes, with operons represented by gray boxes. **b** Nucleotide sequences of the *VC1820* promoter and *VC1822–VC1823* intergenic regions. The ManR-binding sequence AATCC is highlighted in bright green. The initiation and stop codons of the indicated genes are colored in red and blue, respectively.

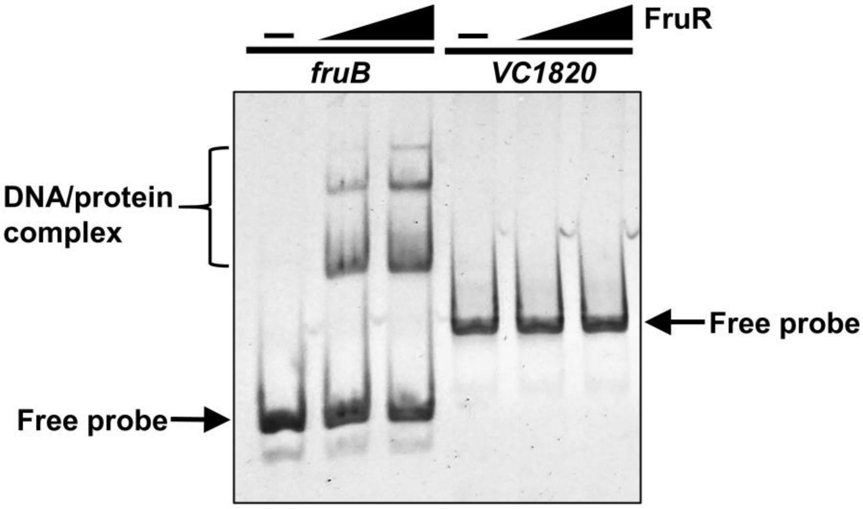

Supplementary Figure 9. FruR does not bind to the *VC1820* promoter

Binding of FruR (0, 120, and 240 ng) to the *VC1820* promoter DNA (60 ng) was tested in comparison with *fruB* promoter DNA (60 ng) as a positive control by EMSA on a 6% polyacrylamide gel in TBE.

Supplementary Table 1. List of genes whose expression significantly decreased in the *manR* deletion mutant compared with WT in mRNA sequencing data analysis

gene	Δ*manR*/Wild-type			Product
	glucose	fructose	mannose	
VC1820	0.219	0.003	0.002	PTS system fructose-specific transporter subunit IIA
VC1825	0.047	0.002	0.003	transcriptional regulator
VC1826	0.457	0.004	0.003	PTS system fructose-specific transporter subunit IIABC
VC1821	0.388	0.005	0.004	PTS system fructose-specific transporter subunit IIBC
VCA0540	0.462	0.468	0.007	formate transporter 1
VC1827	0.693	0.088	0.008	mannose-6-phosphate isomerase
VC1822	0.357	0.070	0.058	PTS system fructose-specific transporter subunit IIABC
VC0539	1.164	0.119	0.070	sulfate ABC transporter permease
VC0540	1.084	0.111	0.074	sulfate ABC transporter permease
VC0538	1.352	0.119	0.076	thiosulfate ABC transporter substrate-binding protein
VC0384	1.219	0.115	0.082	sulfite reductase (NADPH) flavoprotein subunit alpha
VC0606	0.634	2.829	0.084	nitrogen regulatory protein P-II
VC0386	1.096	0.135	0.085	phosphoadenosine phosphosulfate reductase
VC0385	1.183	0.114	0.090	sulfite reductase subunit beta
VC1590	0.847	0.449	0.092	acetolactate synthase
VC1235	1.390	0.168	0.096	sodium/dicarboxylate symporter
VC2558	1.201	0.127	0.106	adenylyl-sulfate kinase
VC1828	0.947	0.611	0.109	hypothetical protein
VC0541	1.060	0.138	0.113	sulfate ABC transporter ATP-binding protein
VC0968	1.267	0.184	0.115	cysteine synthase A
VC1823	0.933	0.111	0.124	PTS system fructose-specific transporter subunit IIB
VC1824	2.405	0.159	0.244	PTS system nitrogen regulatory subunit IIA

Supplementary Table 2. Bacterial and plasmids used in this study

Strains or plasmids	Genotypes and/or Descriptions	References or Source
**Strains**		
Vibrio cholerae		
O1 El Tor N16961	Wild type, Clinical strain	
Δ*manP*	N16961 Δ*VC1826*	This study
Δ*fruA*Δ*manP*	N16961 Δ*fruA*Δ*manP*	This study
Δ*manR*	N16961 Δ*VC1825*	This study
Δ*lacZ*	N16961 Δ*lacZ*	Lab stock
Δ*lacZ*Δ*manR*	N16961 Δ*lacZ*Δ*VC1825*	This study
Δ*crr*	N16961 Δ*crr*	1
Escherichia coli		
ER2566	F$^-$ λ$^-$ *fhuA2* [*lon*] *ompT lacZ*::T7 gene 1 *gal sulA*11 Δ(*mcrC*-*mrr*)114::IS10 R(*mcr*-73::miniTn10-TetS)2 R(*zgb*-210::Tn10) (TetS) *endA1* [*dcm*]	New England Biolabs
Rosetta (DE3) /pLysSRARE	F$^-$ *ompT hsdS*$_B$(rB$^-$ mB$^-$) *gal dcm* (DE3) pLysSRARE(Cm^r)	Novagen
SM10/λpir	*thi thr leu tonA lacY supE recA*::RP4-2-Tc::Mu (λpir R6K) Km^r	2
**Plasmids**		
pDM4	Suicide vector for homologous recombination into *V. cholerae* chromosome, OriR6K, Cm^r	3
pETDuet1		Novagen
pJK1113	pBAD24 with *oriT* of RP4 and *nptl*, P$_{BAD}$; Km^r, Amp^r	4
pBR322	A low copy number cloning vector; Cm^r Tet^r	5
pBAD-MycHisA		Invitrogen
pDM4-*VC1826*	pDM4-based suicide vector for deletion of *manP*, Cm^r	This study
pDM4-*VC1825*	pDM4-based suicide vector for deletion of *VC1825*, Cm^r	This study
pVC1826	pBR322-based *VC1826* expression vector under control of its own promoter, Amp^r	This study
pManR	pBAD-MycHisA-based expression vector for VC1825, Amp^r	This study
pJK-LacZ	pJK1113-based expression vector containing promoter-less LacZ, Amp^r	6
pJK-P$_{manR}$::LacZ	pJK1113-based expression vector for LacZ under control of *manR* promoter, Amp^r	This study
pJK-P$_{manR\mathrm{BS1mut}}$::LacZ	pJK-P$_{manR}$::LacZ based expression vector containing *manR* promoter having mutated BS1, Amp^r	This study
pJK-P$_{manR\mathrm{BS2mut}}$::LacZ	pJK-P$_{manR}$::LacZ based expression vector containing *manR* promoter having mutated BS2, Amp^r	This study

pJK-P$_{\mathit{manR}\mathrm{BS12mut}}$::LacZ	pJK-P$_{\mathit{manR}}$::LacZ based expression vector containing *manR* promoter having mutated BS1 and BS2, Amp^r	This study
pJK-P$_{\mathit{manP}}$::LacZ	pJK1113-based expression vector for LacZ under control of *manP* promoter, Amp^r	This study
pJK-P$_{\mathit{manP}\mathrm{BS1mut}}$::LacZ	pJK-P$_{\mathit{manP}}$::LacZ based expression vector containing *manP* promoter having mutated BS1, Amp^r	This study
pJK-P$_{\mathit{manP}\mathrm{BS2mut}}$::LacZ	pJK-P$_{\mathit{manP}}$::LacZ based expression vector containing *manP* promoter having mutated BS2, Amp^r	This study
pJK-P$_{\mathit{manP}\mathrm{BS12mut}}$::LacZ	pJK-P$_{\mathit{manP}}$::LacZ based expression vector containing *manP* promoter having mutated BS1 and BS2, Amp^r	This study
pETDuet1-VC1825	pETDuet1-based expression vector for VC1825, Amp^r	This study
pETDuet1-His-VC1825	pETDuet1-based expression vector for N-terminal His$_6$ tagged VC1825, Amp^r	This study
pETDuet1-VC1825-His	pETDuet1-based expression vector for C-terminal His$_6$ tagged VC1825, Amp^r	This study
pET43.1a-FruR	pET43.1a-based expression vector for FruR, Amp^r	[6]
pET24a-Mlc	pET-24a-based expression vector for Mlc, Km^r	[7]
pET43.1a-CRP	pET43.1a-based expression vector for CRP, Amp^r	Lab stock

*Cm^r, chloramphenicol-resistant; Amp^r, ampicillin-resistant; Km^r, kanamycin-resistant; Tet^r, tetracycline-resistant.

Supplementary Table 3. Oligonucleotides used in this study

Name	Nucleotides sequence (5’-3’)	Uses
VC1826-d1F	TCTCCACCTCTAGACAATTCTAAGCGTGGAAAATTCACC	Construction of pDM4-based vector for in-frame deletion
VC1826-d1R	ATAACGAGGAAGGGAGAAAGAGACCTCTGTACATTTTATTTTTATGTC	
VC1826-d2F	TAAAATGTAC AGAGGTCTCTTTCTCCCT TCCTCGTTAT GGCTTGAGGT	
VC1826-d2R	GCGCATAAACCACTCGAGGTTTGTGATTGGGGTCTTTGTAATTTCG	
VC1825-d1F	AAAAAATCTAGAGAATGCCGCTTTATCTGAAATTC	
VC1825-d1R	TGAAGCACGATAATCATGTAAACCCTCACTTCGTC	
VC1825-d2F	AGTGAGGGTTTACATGATTATCGTGCTTCAATTTA	
VC1825-d2R	TTTTTTCTCGAGATGAGTTCTCTAGCCTGCTCGCC	
PZ-F	ATCAAAGTCGACATGTAAACCCTCACTTCG	Construction of pJK-P_{manP}::LacZ
PZ-R	TTGGTTGTCGACAGAGACCTCTGTACATTT	
RZ-F	TTGGTTGTCGACAGAGACCTCTGTACATTT	Construction of pJK-P_{manR}::LacZ
RZ-R	ATCAAAGTCGACATGTAAACCCTCACTTCG	
BS1mut-F	GAGCAACATCACACTCAAGACAGAATGATACAAT CACTA AGTTTTAACACCAATTCTC	Introduction of mutated sequence into *manR-manP* intergenic region
BS1mut-R	GGTTTGTGCTAGAGAATTGGTGTTAAAACTTAGTGATTGTATCATTCTGTCTTGAG	
BS2mut-F	CGAGATCTAAATCACACCAAACCAATAGAGTTAC CACTA TCTAGTGAATGTTTTATTTCTGC	
BS2mut-R	CTTATTAGTGCAGAAATAAAACATTCACTAGATAGTGGTAACTCTATTGGTTTGGTGTG	
pBRVC1826-F	CATCAAAGGATCCATGTAAACCCTCACTTCGTCATCGCACT	Construction of pBR322-VC1826
pBRVC1826-R	ACGAGGAAGCTCGAGTTAAGCCGCAGCGGCTTCAAACTTT	
pBADVC1825-F	GAAGTGAGGGCTCGAGTATGATATTTGATGATTTG	Construction of VC1825 expression plasmid
pBADVC1825-R	GCGATGTCAGGTACCTTAAATTGAAGCACGATAAT	
VC1825-F	GAAGTGAGGGAGATCTTATGATATTTG ATGATTTG	
VC1825-R	CGCGATGTCACTCGAGTTAAATTGAAGCACGATA	
HisVC1825-F	AAAAAAAGGATCCAATGATATTTGATGATTTG	
HisVC1825-R	AAAAAAGTCGACTTAAATTGAAGCACGATAATCCG	
VC1825His-F	GAAGTGA GGGAGATCTTATGATATTTG ATGATTTGAT TTCCTCGGTG	
VC1825His-R	TTTTTTTCTCGAGTTAGTGGTGATGATGGTGATG	

	AATTGAAGCACGATAATCCGTTGG	
399-bp-F	ATCAAATATCATATGTAAACCCTCACTTCG	
399-bp-R	TCAATTGGTTGATCATAGAGACCTCTGTAC	
100-bp-1F	CATCAAATATCATATGTAAACCCTC ACTTCG	
100-bp-1R	GAGTGTGATGTTGCTCAAAGAGACT	
100-bp-2F	GTCAGATATACCCCGAATGCCATTG	
100-bp-2R	TTGTGCTAGAGAATTGGTGTTAAAACTGG	
100-bp-3F	AAGACAGAATGATACAATAATCCAG TTTTAAC	
100-bp-3R	ATTAATTTTCATTTAGTTATATTATGTTTGTTGTTATTCATGG	
100-bp-4F	ACCTCCACCATGAATAACAACAAAC	
100-bp-4R	TCTATTGGGGTAGTGAGGCCAATTT	
100-bp-5F	GAGTTAAACCTACAATCATTACTTA AAATTGGCC	
100-bp-5R	ACTAGAGGATTGTAACTCTATTGGTTTGG	
100-bp-6F	CGCTACGAGATCTAAATCACACCAA	
100-bp-6R	TTATTCTTTAATTTTTTTATTTTTCTTATTAGTGCAGAAATAAAAC	
100-bp-7F	GAATGTTTTATTTCTGCACTAATAAGAAAAATAAAAAAATT	
100-bp-7R	TCAATTGGTTGATCATAGAGACCTCTG	
MB1-F	AGACAGAATGATACAATAATCCAGTTTTAACACCAATTC	
MB1-R	GAATTGGTGTTAAAACTGGATTATTGTATCATTCTGTCT	
MB1-null-F	AGACAGAATGATACAATAGTTTTAACACCAATTC	EMSA probe
MB1-null-R	GAATTGGTGTTAAAACTATTGTATCATTCTGTCT	
MB1-mut-F	AGACAGAATGATACAATCACTAAGTTTTAACACCAATTC	
MB1-mut-R	GAATTGGTGTTAAAACTTAGTGATTGTATCATTCTGTCT	
MB2-F	CAAACCAATAGAGTTACAATCCTCTAGTGAATGTTTTAT	
MB2-R	ATAAAACATTCACTAGAGGATTGTAACTCTATTGGTTTG	
MB2-null-F	CAAACCAATAGAGTTACTCTAGTGAATGTTTTAT	
MB2-null-R	ATAAAACATTCACTAGAGTAACTCTATTGGTTTG	
MB2-mut-F	CAAACCAATAGAGTTACCACTATCTAGTGAATGTTTTAT	
MB2-mut-R	ATAAAACATTCACTAGATAGTGGTAACTCTATTGGTTTG	
nagE-F	GCAAGCTGCTTCACTTCAATC	
nagE-F	CTTAAGTTCCCCCTATAGGATTTTTGTATAG	
ptsH-F	TACGTGGTTAAATCAAATTTTCGTTTAAAAAAGC	
ptsH-R	GTTTTATACCCCAATGAGTTTATTTTTTGTAAGTTTG	
fruB-F	AAGGGGGTCTCGTTTTATGTGCGTC	
fruB-R	TCTTAACTCCTGTCTGCCTC TATAG	
VC1820-F	CTCGGTTTCTTCTAAAAGCTTAATATTCAG	

VC1820-R	AGCTCGGTGATCATTTATTGGCTCAGAAAG	
VC1822-23-F	TGGGTAATATCCACAATTGTTTTACCTTAC	
VC1822-23-F	CGCAACAATTTTCATGGAAATCCCTCATTG	
rrsG-qRT-F	TTAGCCGGTGCTTCTTCTGT	qRT-PCR
rrsG-qRT-R	CAGCCACACTGGAACTGAGA	
1826-qRT-F	CCAAGGGCGAATTTCAGATA	
1826-qRT-R	TTGGAAGGCAAACCATCTTC	
1825-qRT-F	TTGCGGGTGATTTTCATACA	
1825-qRT-R	CGGTATCCCAGTTCGGTTTA	
1827-qRT-F	ACTCGAAAACCCAAACGATG	
1827-qRT-R	GCCGCCAAAATTTTAAACAA	
1820-qRT-F	TGGCGCAAATGTTACTTGAG	
1820-qRT-R	CGGTTTGATGCCTTCTTCAT	
1821-qRT-F	ACGTTCTGCTTCATGCTCCT	
1821-qRT-R	CTTCTGCGATTTCGTCTTCC	
1822-qRT-F	ATCGCTCAAATGGGTATTGC	
1822-qRT-R	TCAGCTGACGAACCACAAAG	
1823-qRT-F	AAGCCGCACCAAAGTACAAT	
1823-qRT-R	CTTCGATCGAGAGCTTCACC	
1824-qRT-R	CACCAAATCTCTCGCGGTAT	
1824-qRT-R	GGCATAGTGCCATTTGACCT	
1F	ATCAAATATCATATGTAAACCCTCACTTCG	RT-PCR
1R	TCAATTGGTTGATCATAGAGACCTCTGTAC	
2F	CCAAGGGCGAATTTCAGATA	
2R	TTGGAAGGCAAACCATCTTC	
3F	CCCTAGTGCCGATCATGATCGCGCC	
3R	CGGAGCAGCCATTAGGATGAGCGCC	
4F	ACTCGAAAACCCAAACGATG	
4R	GCCGCCAAAATTTTAAACAA	
NGSP-*manP*-R	CCGCTTTATCTGAAATTCGCCCTTGGGC	5′RACE
GSP-*manP*-R	GGCAAACCATCTTCTGCGCCATAGT	
TSS-F1	CCAATAGAGTTACAATCCTCTAGTGA	Confirmation of *manP* TSS
TSS-F2	TTCTGCACTAATAAGAAAAATAAAAAAATTAAAGAATAACTCT	
TSS-F3	ACATAAAAATAAAATGTACAGAGGTCTCT	
TSS-F4	CCAAGGGCGAATTTCAGATA	
TSS-R	TTGGAAGGCAAACCATCTTC	
footprinting-F	AATGATTCACG CTCATTAAGCACCGAGGAAA	DNase I footprinting
footprinting-R	GCTTGCAGATCAAGCTGAATTAAATCGGCA	

*Engineered restriction sites were underlined.

Supplementary References

1. Heo, K. *et al.* Sugar-mediated regulation of a c-di-GMP phosphodiesterase in *Vibrio cholerae*. *Nat Commun* **10**, 5358, doi:10.1038/s41467-019-13353-5 (2019).
2. Miller, V. L. & Mekalanos, J. J. A novel suicide vector and its use in construction of insertion mutations: osmoregulation of outer membrane proteins and virulence determinants in *Vibrio cholerae* requires toxR. *J Bacteriol* **170**, 2575-2583, doi:10.1128/jb.170.6.2575-2583.1988 (1988).
3. Milton, D. L., O'Toole, R., Horstedt, P. & Wolf-Watz, H. Flagellin A is essential for the virulence of *Vibrio anguillarum*. *J Bacteriol* **178**, 1310-1319, doi:10.1128/jb.178.5.1310-1319.1996 (1996).
4. Lim, J. G., Bang, Y. J. & Choi, S. H. Characterization of the *Vibrio vulnificus* 1-Cys peroxiredoxin Prx3 and regulation of its expression by the Fe-S cluster regulator IscR in response to oxidative stress and iron starvation. *J Biol Chem* **289**, 36263-36274, doi:10.1074/jbc.M114.611020 (2014).
5. Sambrook, J., Fritsch, E. F. & Maniatis, T. *Molecular cloning: a laboratory manual.* (Cold spring harbor laboratory press, 1989).
6. Yoon, C. K., Kang, D., Kim, M. K. & Seok, Y. J. *Vibrio cholerae* FruR facilitates binding of RNA polymerase to the fru promoter in the presence of fructose 1-phosphate. *Nucleic Acids Res* **49**, 1397-1410, doi:10.1093/nar/gkab013 (2021).
7. Yoon, J. H., Jeon, M. S., Eyun, S. I. & Seok, Y. J. Evidence for reciprocal evolution of the global repressor Mlc and its cognate phosphotransferase system sugar transporter. *Environ Microbiol* **24**, 122-136, doi:10.1111/1462-2920.15803 (2022).
